# Widespread influence of artificial light at night on ecosystem metabolism

**DOI:** 10.1038/s41558-025-02481-0

**Published:** 2025-11-12

**Authors:** Alice S. A. Johnston, Jiyoung Kim, Jim A. Harris

**Affiliations:** Cranfield Environment Centre, Cranfield University, Cranfield, UK

**Keywords:** Biogeochemistry, Ecophysiology

## Abstract

Artificial light pollution is increasing worldwide with pervasive effects on ecosystem structure and function, yet its influence on ecosystem metabolism remains largely unknown. Here we combine artificial light at night (ALAN) intensity metrics with eddy covariance observations across 86 sites in North America and Europe to show that ALAN indirectly decreases annual net ecosystem exchange by enhancing ecosystem respiration (*R*_e_). At half-hourly and daily scales, we detect consistent nonlinear interactions between ALAN and night duration, with *R*_e_ increasing under higher ALAN and partially decoupling from gross primary production. At the annual scale, gross primary production shows no direct ALAN response and is instead influenced by the growing season length and urban proximity, whereas *R*_e_ responds more strongly and consistently across timescales. Our findings show that ALAN disrupts the fundamental energetic constraints on ecosystem metabolism, warranting the inclusion of light pollution in global change and carbon–climate feedback assessments.

## Abstract (web summary)

The authors combine light intensity data with eddy covariance observations from 86 sites to show that artificial light at night increases ecosystem respiration and alters carbon exchange, with impacts shaped by diel cycles and seasonal dynamics.

## Main

Artificial light pollution is accelerating across the globe^1,2^ and has widespread consequences for people^3,4^ and the planet^5–7^. Shifts in the luminance and spectral composition of the nocturnal environment modify the physiology, behaviour and ecological interactions of organisms^7–11^, which together play a fundamental role in ecosystem metabolism^12,13^. Ecosystem metabolism, comprising gross primary production (GPP) and ecosystem respiration (*R*_e_), directs the magnitude and direction of carbon–climate feedbacks via net ecosystem exchange (NEE)^14^. Around one quarter of global terrestrial ecosystems are exposed to artificial light at night (ALAN)^15^, but the effects on ecosystem metabolism are currently unknown.

Changing daily and seasonal cycles of light and dark^10^ could decouple the timing of biological processes across trophic networks^16^. Trophic groups are also exposed to ALAN at different intensities and have varying sensitivities to luminance and spectral composition^17^. Plant responses to photoperiod are influenced even at low ALAN intensities^18,19^, and longer-term exposure influences seasonal phenology, growth form, resource allocation and, thus, potentially carbon fixation^20^. High ALAN intensity exposure in urban areas disrupts the behavioural patterns of nocturnally migrating birds^21^ and plant diversity^22^ and restructures soil microbial communities, reducing the functional genes involved in nutrient regulation and plant health^23^. Together, the observed effects of ALAN across levels of biological organization and diverse taxa suggest a potential cascading impact on ecosystem structure and function. Previous studies of ALAN effects, however, have focused on local or experimental manipulations, leaving uncertainty about whether ALAN effects persist at the ecosystem level and longer timescales.

GPP and *R*_e_ are fundamentally constrained by shortwave (solar) radiation (SW) and temperature (*T*), respectively^24–26^. That is, SW determines the direction and duration of energy flow between the atmosphere and ecosystems, and *T* determines the rate of reactions^12^. Although ALAN is not expected to influence SW or *T* directly, artificial light could disrupt the processing of energy according to these fundamental constraints via acclimation, compensation and adaptation strategies^27,28^. A better understanding of the magnitude and direction of ALAN effects on ecosystem metabolism could help constrain carbon–climate processes in Earth system models (ESMs)^29^. Specifically, largely uncertain ESM processes and their response to climatic factors could be compounded by the chronic effects of pervasive anthropogenic stressors, such as ALAN.

**Fig. 1:**
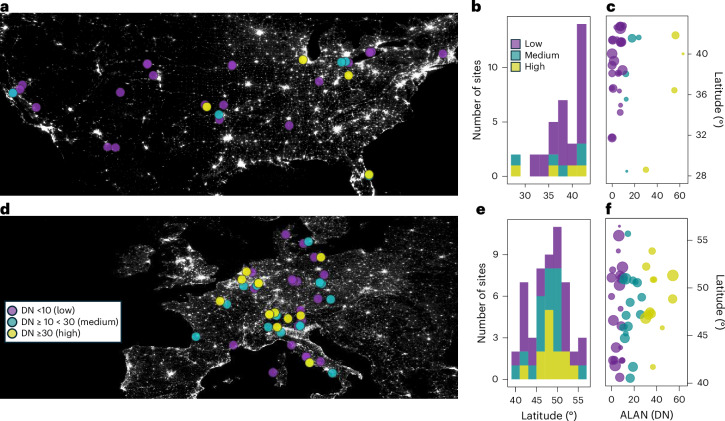
Distribution of flux tower sites across artificial light intensity in North America and Europe. **a**,**d**, The location of 86 eddy covariance flux tower sites from FLUXNET2015 (symbols, colours indicate ALAN intensity according to DN (higher values represent greater luminance of light at night) (as in **d**) displayed over a harmonized global nighttime light map for 2012 (for visualization only) in North America (*n* = 34) (**a**) and Europe (*n* = 52) (**d**). **b**,**e**, The latitudinal distribution of sites with different ALAN intensities for North America (**b**) and for Europe (**e**), in 2° N intervals. **c**,**f**, The ALAN intensities of selected FLUXNET2015 sites, averaged across site years (the number of years with observational data per site), for North America (**c**) and Europe (**f**) according to DN with symbol size indicating number of site years (range: 1–20 years per site between 1992 and 2014, total site years in **c** is 211 and in **f** is 412). Basemaps in **a** and **d** were generated with QGIS using the harmonized global nighttime light dataset^32^ under a Creative Commons license CC BY 4.0.

**Extended Data Fig. 1:**
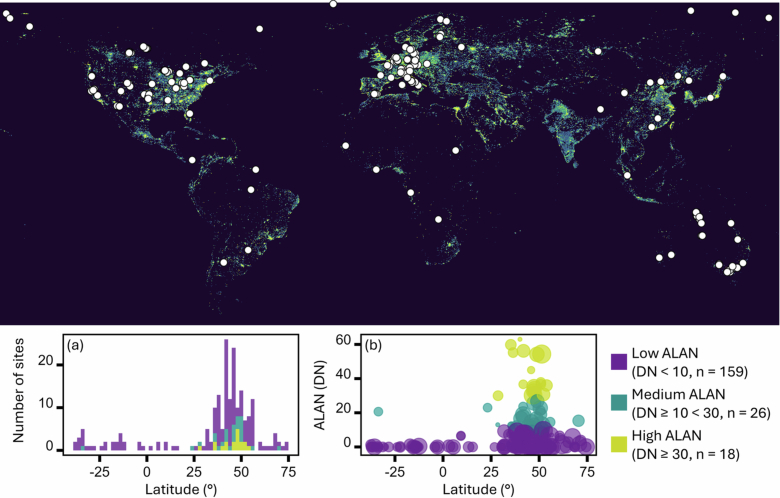
Global distribution of FLUXNET2015 sites across artificial light intensity gradients. White symbols indicate FLUXNET2015 sites within the latitudinal range of the harmonised nighttime light map (n = 203, see Methods). Panel **a**) shows number of sites and **b**) shows artificial light at night (ALAN) digital number (DN) averaged over site years, plotted against latitude. Bar and symbol colours in **a**) and **b**) indicate low (DN < 10), medium (DN ≥ 10 ≤ 30) and high (DN > 30) ALAN intensities. Sites for analysis in this study were selected based on replication of FLUXNET2015 sites across high, medium and low ALAN intensities (Fig. 1 in the main text, see Methods). Basemap generated with QGIS using the harmonized global nighttime light dataset^32^ under a Creative Commons license CC BY 4.0.

Global efforts to measure carbon exchange across diverse ecosystems^30^ combined with satellite observations of ALAN distribution and intensity across the land surface^2,31^ enable the exploration of artificial light’s influence on terrestrial ecosystem metabolism. Here, we leverage the harmonized nighttime light dataset of Li et al.^32^ and eddy covariance observations from FLUXNET2015^30^ to investigate the instantaneous and aggregated influence of ALAN on ecosystem-scale NEE, GPP and *R*_e_ fluxes. Although both datasets have global coverage, the location of eddy covariance flux towers are biased towards dark sky regions (Extended Data Fig. 1). Following definitions by Li et al.^32^ and others^33^, we use three digital number (DN, higher values represent greater luminance of light at night; Methods) groups representative of low (DN <10), medium (DN ≥ 10 < 30) and high (≥30, representative of urban boundaries) ALAN intensity to identify regions with FLUXNET2015 sites across a range of ALAN intensities. North America and Europe were the only regions, globally, with more than one high ALAN intensity FLUXNET2015 site (Methods; Fig. 1a,d). Within both North America and Europe, sites were selected on the basis of latitudinal ranges at which medium or high ALAN intensity sites were present (Fig. 1b,e) to minimize climatic factors in higher or lower latitude sites being ascribed to low ALAN intensities. In total, 86 FLUXNET2015 sites were selected, 34 sites in North America (4, 5 and 25 sites at high, medium and low ALAN intensities, respectively) and 52 sites in Europe (13, 17 and 22 sites at high, medium and low ALAN intensities, respectively) (Methods; Fig. 1 and Supplementary Table 1). Despite regional imbalances in FLUXNET2015 site distribution across ALAN intensity levels, the dataset captures a diverse range of ALAN intensities across temperate regions experiencing similar seasonal fluctuations in *T* and SW.

To detect the potential influence of ALAN on ecosystem metabolism, we investigate half-hourly and mean daily ecosystem carbon fluxes (*F*_c_; *F*_c_: NEE, GPP and *R*_e_) measurements against their fundamental constraints, *T* and SW, according to the modified Arrhenius equation of Weyhenmeyer^12^:

1 $${F}_{{\rm{c}}}={T}^{4}\sigma {{\rm{e}}}^{\frac{-\mathrm{SW}}{\sigma {T}^{4}}}{k}_{{F}_{{\rm{c}}}}-b,$$

where *F*_c_ is ecosystem C flux (NEE, GPP, *R*_e_) (in µmol CO^2^ m^−2^ s^−1^), *T* is temperature in Kelvin, *σ* is the Stefan–Boltzmann constant (in J m^−2^ s^−1^ K^−4^) (5.67 × 10^−8^), SW is incoming SW (in J m^−2^ s^−1^,) *k*_*F*__c_ is the slope of the linear relationship and *b* is the intercept. The function establishes a biophysically grounded baseline for different *F*_c_ by capturing their shared fundamental constraints (*T* and SW). The use of the modified Arrhenius function in this study primarily serves as a comparative baseline rather than a mechanistic model, enabling deviations attributable to chronic ALAN effects to be identified relative to fundamental energetic constraints.

**Fig. 2:**
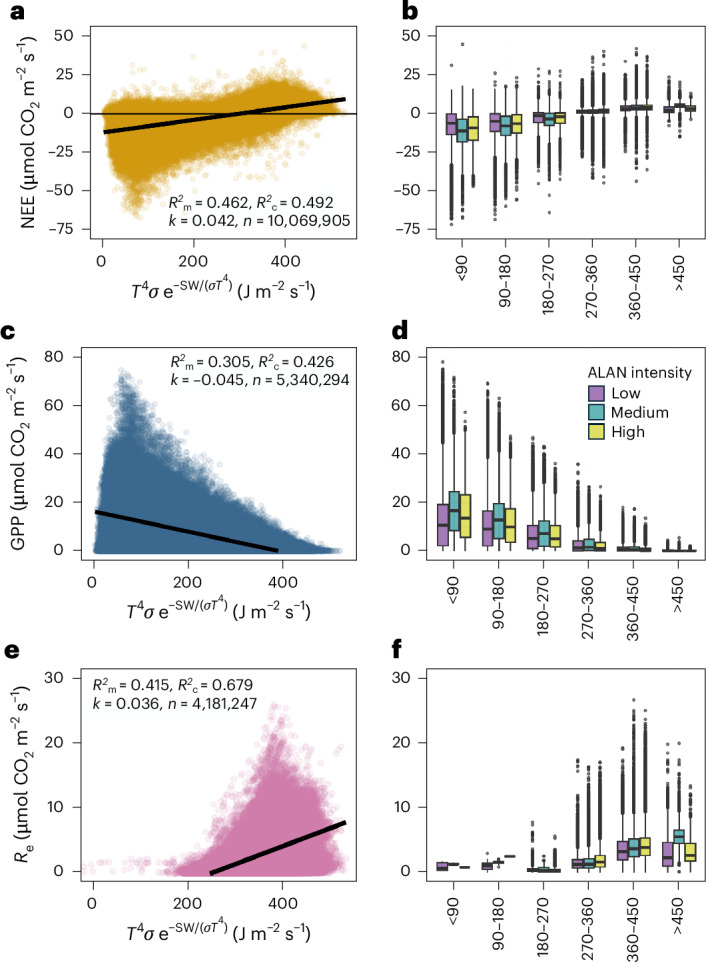
Ecosystem carbon flux dependence on modified Arrhenius constraints and the effect of ALAN. **a**,**c**,**e**, The symbols are half-hourly FLUXNET2015 measurements for NEE (gold symbols) (**a**), daytime GPP (blue symbols) (**c**) and nighttime *R*_e_ (magenta symbols) (**e**) for 86 sites across North America and Europe. The linear regression lines in **a**, **c** and **e** indicate fixed-effect relationships of fundamental constraints on ecosystem carbon fluxes according to the modified Arrhenius function (null models as in equation (1)). **b**,**d**,**f**, The box plots display the distribution of measured fluxes across bins of the modified Arrhenius function (axes labels and units in **b**, **d** and **f** are the same as in **a**, **c** and **e**, respectively) grouped by ALAN intensity to illustrate variation in carbon fluxes relative to energetic constraints as a function of ALAN. The boxes represent interquartile ranges (IQR), the horizontal lines denote medians, the whiskers extend to 1.5 × IQR and the points indicate outliers.

The null models for NEE, GPP and *R*_e_ were linear mixed effect models (LMMs) or generalized additive mixed models (GAMMs) fitted to equation (1) (Methods) with FLUXNET2015 site and latitude as random effects and fundamental constraint (${T}^{4}\sigma \,{{\rm{e}}}^{\frac{-\mathrm{SW}}{\sigma {T}^{4}}}$, J m^−2^ s^−1^) as a fixed effect (Fig. 2). The null models were tested against models with additional explanatory variables, including continent, climate, International Geosphere–Biosphere Programme land use classifications, growing season (GS), night duration (ND, hours), vapour pressure deficit (VPD, hectopascals), precipitation (P, millimetres), ALAN intensity (DN), distance to nearest urban polygon (DtNUP, kilometres) and proportion of urban land cover in 3- and 10-km buffers around each site (pULC_3km, pULC_10km). The LMM selection criteria for explanatory variables followed a trade-off between explanatory power and parsimony, with the condition that additional degrees of freedom (df) were accompanied by lower Akaike information criteria (AIC) and higher marginal *R*^2^ (*R*^2^_m_) goodness-of-fit measures (ΔAIC_df_ <−5 and Δ*R*^2^_mdf_ >0.01 compared with the null model (Methods; Fig. 2a,c,e). All *F*_c_ LMMs selected GS; GPP and *R*_e_ LMMs selected ND; the GPP LMM selected DtNUP; and the *R*_e_ LMM selected VPD and ALAN (Supplementary Tables 2 and 3).

**Fig. 3:**
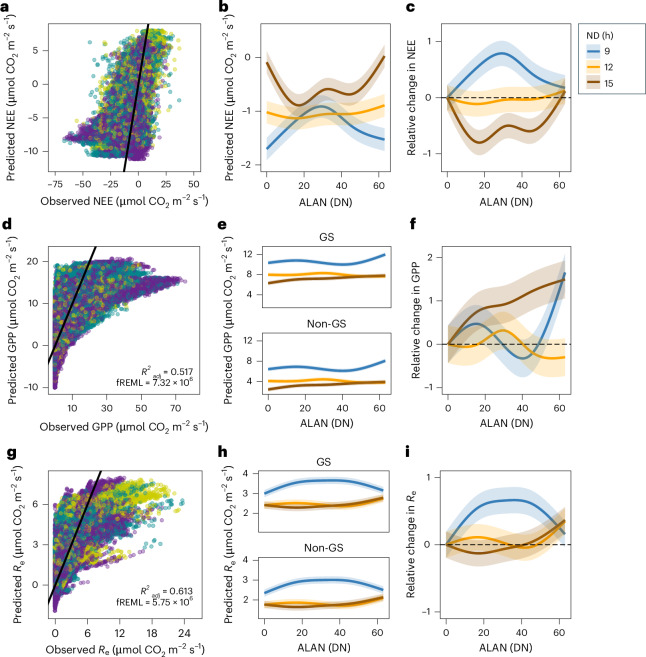
Nonlinear influence of ALAN and ND on ecosystem metabolism. **a**–**i**, GAMMs fitted to half-hourly carbon flux measurements consistently selected smooth tensor product interactions between ALAN and ND for NEE (**a**–**c**), GPP (**d**–**f**) and *R*_e_ (**g**–**i**). **a** (*R*^2^_adj_ = 0.430; fREML = 1.35 × 10^7^), **d** and **g** show observed versus predicted fluxes for the final variance-weighted GAMMs (Supplementary Table 5), which account for heteroscedasticity across the range of observed fluxes (Supplementary Table 4 and Extended Data Fig. 2). **b**, **e** and **h** illustrate smooth estimates of the tensor product interaction across the gradient of ALAN intensity and ND (coloured lines, with shaded ribbons representing mean predictions ± 95% confidence intervals). For GPP, **e**, and *R*_e_, **h**, predictions are shown separately for the GS and non-GS, whereas for NEE, **b**, the GS was not selected as a significant predictor. **c**, **f** and **i** depict GAMM-derived estimates of the relative change in each flux across gradients of ALAN intensity and ND, expressed relative to ALAN = 0, with shaded areas denoting 95% confidence intervals around the mean.

Backward selection and variance-weighting were applied to GAMMs fitted to half-hourly NEE, GPP and *R*_e_ observations (Methods; Supplementary Table 4) with the explanatory variable identified in the LMMs. In the final GAMMs, all *F*_c_ retained a significant interaction between ALAN and ND (Fig. 3 and Supplementary Table 5). Notably, the NEE GAMM did not retain GS as a significant predictor, suggesting that the seasonality in instantaneous NEE responses was captured by GPP and *R*_e_, which both retained GS effects (Supplementary Table 5). During GAMM selection, VPD (selected in the *R*_e_ LMM) exhibited consistently high concurvity (>0.8) with other smooth terms, including models in which ALAN was removed, indicating substantial collinearity with the modified Arrhenius function. DtNUP, selected in the GPP LMM, contributed no additional explanatory power in all GAMMs.

**Extended Data Fig. 2:**
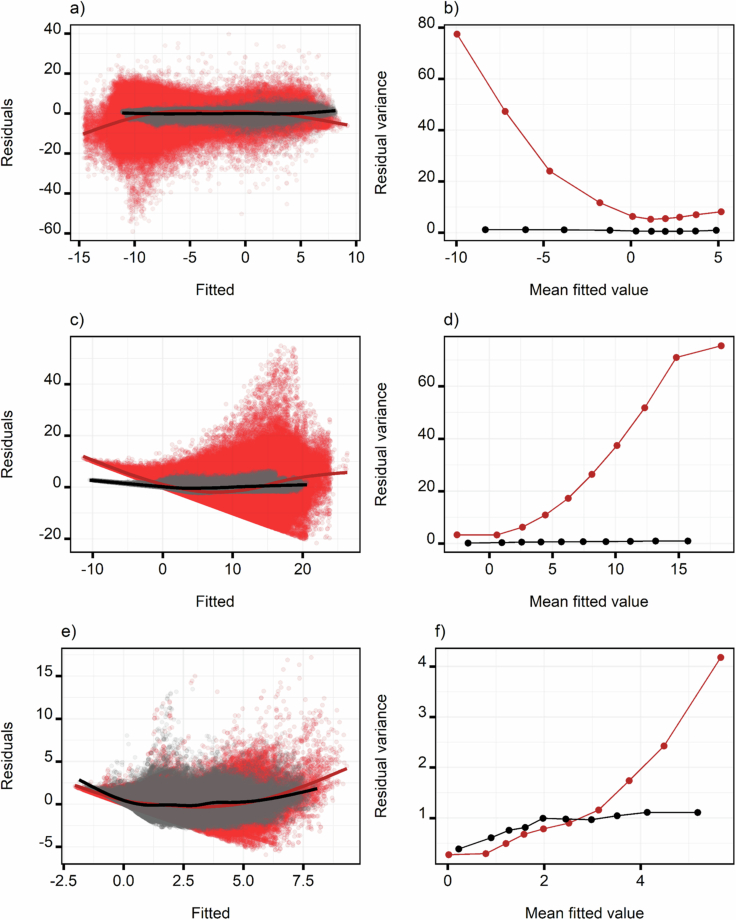
Residual diagnostics for unweighted and weighted GAMMs of half-hourly carbon fluxes. Final unweighted GAMMs (red symbols and lines) and weighted GAMMs (black symbols and lines) were fitted to half-hourly flux observations (see Supplementary Table 4) for **a, b**) *NEE*, **c, d**) *GPP*, **e, f**) *R*_e_. The left-hand panel (**a, c, e**) show GAMM residuals plotted against mean fitted values, with fitted loess lines. The right-hand panel (**b, d, f**) show residual variance by fitted value bins, indicating a reduction in heteroscedasticity across the range of predicted values in the weighted GAMMs (black symbols and lines), presented in Fig. 3 and Supplementary Table 5.

**Extended Data Fig. 3:**
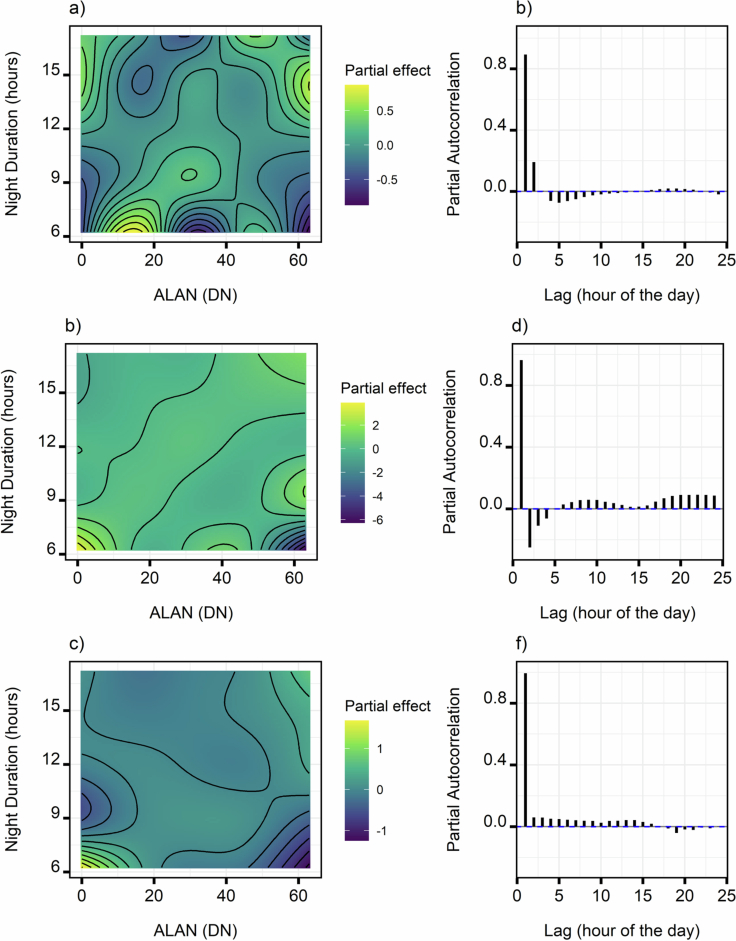
Partial effect surfaces and residual autocorrelation for half-hourly GAMMs. Final weighted half-hourly GAMMs for **a, b**) *NEE*, **c, d**) *GPP* and **e, f**) *R*_e_ are shown. Partial effect surfaces (**a, c, e**) display the estimated tensor-product smooth interaction between ALAN and night duration, as derived from the final GAMMs in Supplementary Table 5. Panels b, d and e show partial autocorrelation functions of the model residuals (at hourly lags), indicating the degree of remaining temporal autocorrelation after model fitting. Dashed blue lines represent approximate 95% confidence intervals for the null hypothesis of white noise.

The variance weighting substantially reduced residual heteroscedasticity across all *F*_c_, with the scale estimate reduced by ~95% and adjusted *R*² (*R*^2^_adj_) reduced by 0.06–0.11 in weighted compared with unweighted final GAMMs, indicating improved model stability through decreased overfitting to high-variance observations (Supplementary Table 4 and Extended Data Fig. 2). Figure 3, right panels, shows weighted GAMM estimates of relative changes in each *F*_c_ across gradients of ALAN intensity and ND. Partial effect surfaces illustrating nonlinear ALAN × ND interactions at half-hourly timescales are presented in Extended Data Fig. 3, along with residual diagnostics indicating no substantial autocorrelation after model fitting.

**Fig. 4:**
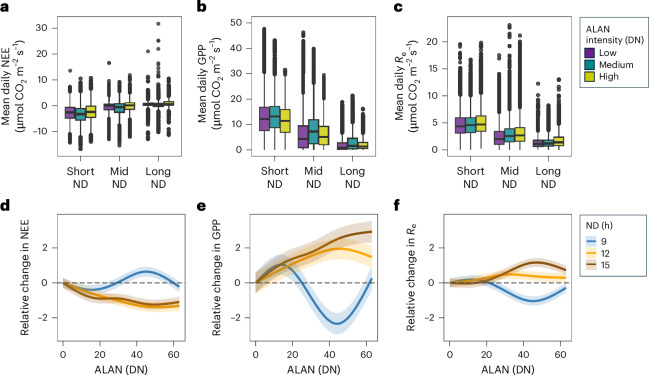
Nonlinear response of mean daily ecosystem carbon fluxes to ALAN and ND. **a**–**c**, The box plots display the distribution of measured mean daily NEE (**a**), GPP (**b**) and *R*_e_ (**c**) across ND groups (short ND: <9.5, mid ND: ≥9.5 < 13.9, long ND: ≥13.9 h, defined by 25% and 75% quartiles) coloured by ALAN intensity (*n* = 197,247). The boxes represent the interquartile ranges (IQR), the horizontal lines denote medians, the whiskers extend to 1.5× the IQR and the points indicate outliers. **d**–**f**, The variance-weighted GAMM predictions (Supplementary Table 9) for relative changes in daily mean NEE (**d**), GPP (**e**) and *R*_e_ (**f**), expressed relative to ALAN = 0 for ND groups as in Fig. 3, with shaded ribbons representing mean predictions ± 95% confidence intervals.

**Extended Data Fig. 4:**
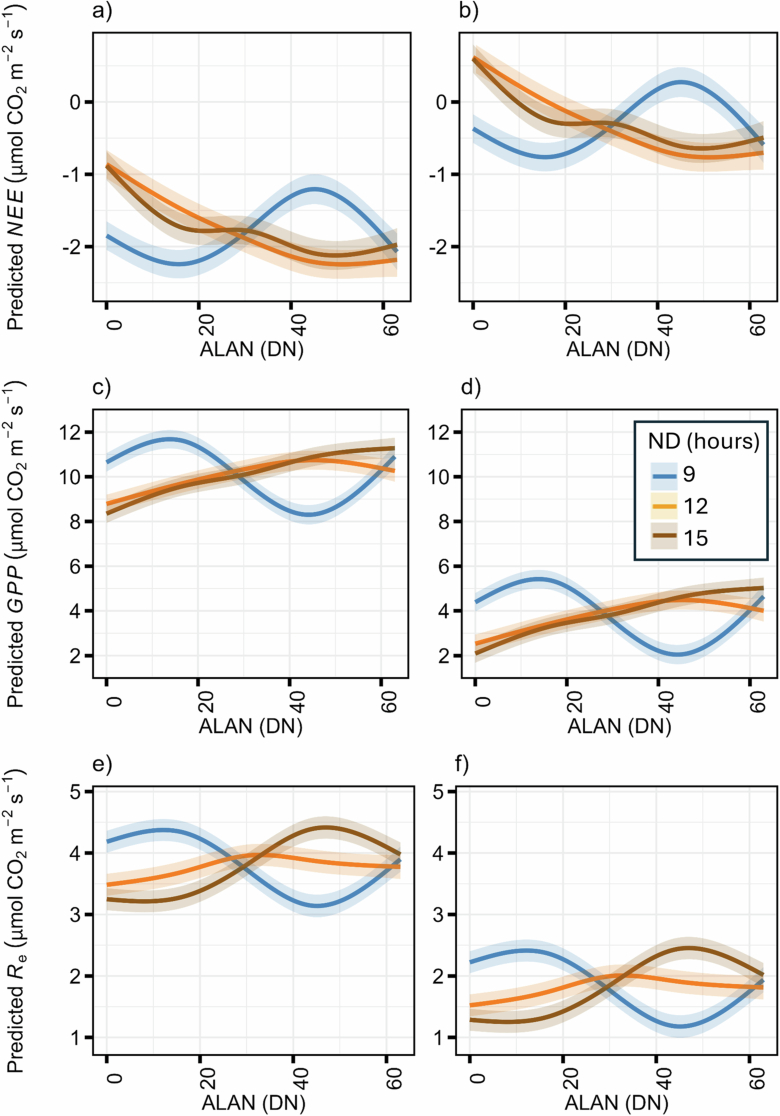
Predicted conditional smooths of daily GAMMs for carbon fluxes during growing and non-growing seasons. Panels show: **a, b**) *NEE*, **c, d**) *GPP* and **e, f**) *R*_e_ in the growing season (**a, c, e**) and non-growing season (**b, d, f**) (Supplementary Table 9). Smooth curves illustrate the tensor-product interaction between ALAN and night duration (ND, coloured lines: 9, 12 and 15 h, with shaded ribbons representing mean predictions ±95% confidence intervals).

**Extended Data Fig. 5:**
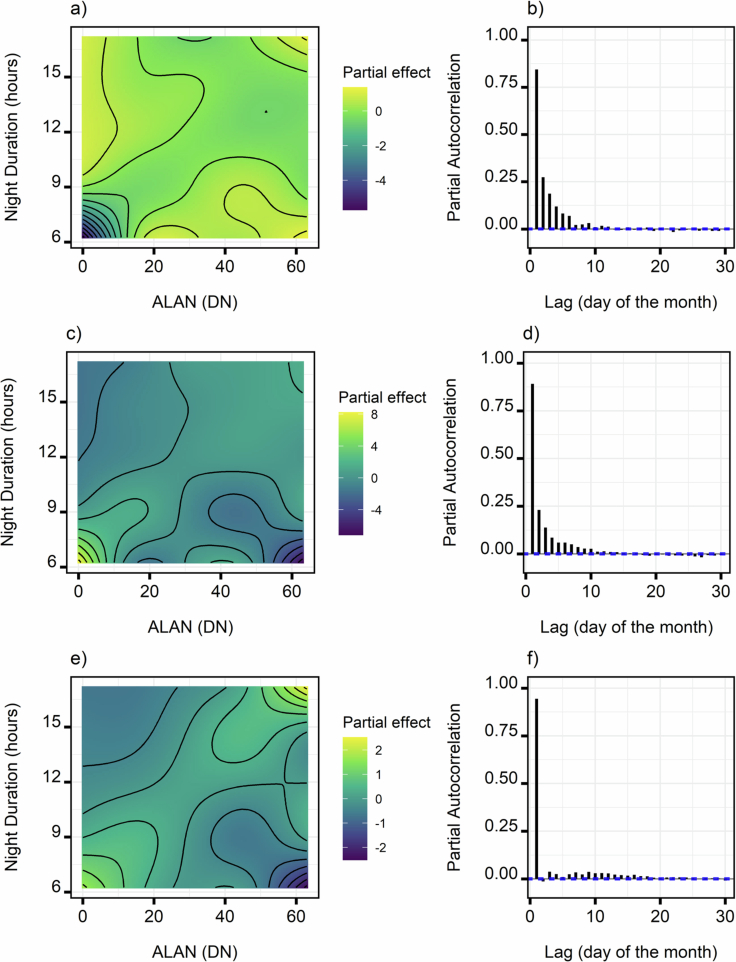
Partial effect surfaces and residual autocorrelation for daily GAMMs of carbon fluxes. Final variance-weighted daily GAMMs for **a, b**) *NEE*, **c, d**) *GPP* and **e, f**) *R*_e_ are shown. Partial effect surfaces (**a, c, e**) display the estimated tensor-product smooth interaction between ALAN and night duration, as derived from the final GAMMs in Supplementary Table 9. Panels **b**, **d** and **e** show partial autocorrelation functions of the model residuals (at day of the month lags), indicating the degree of remaining temporal autocorrelation after model fitting. Dashed blue lines represent approximate 95% confidence intervals for the null hypothesis of white noise.

LMMs and GAMMs fitted to mean daily NEE, GPP and *R*_e_ yielded more consistent trends compared with models fitted to half-hourly observations. All daily LMMs and GAMMs identified GS and ND as significant predictors, with the GPP LMM selecting DtNUP and the *R*_e_ LMM selecting ALAN as explanatory variables (Supplementary Tables 6 and 7). All of the daily GAMMs selected the smooth tensor product between ALAN and ND (Supplementary Tables 8 and 9). Compared with the half-hourly models, the daily GAMMs exhibited smoother and more monotonic relationships between ALAN and *F*_c_, reflecting the reduction in diel and short-term variability through temporal aggregation (Extended Data Fig. 4). Temporal aggregation led to clearer trends in predicted relative changes in *F*_c_ across gradients of ALAN intensity (Fig. 4), in contrast to more variable patterns in the half-hourly GAMM predictions (Fig. 3c,f,i). Notably, whereas ALAN consistently increased *R*_e_ in half-hourly GAMMs and particularly during short nights (Fig. 3h,i), the daily GAMMs showed a contrasting pattern, with *R*_e_ increasing most with ALAN intensity during longer nights (Fig. 4c,f). This divergence demonstrates how the aggregation of diel variability can modify the apparent direction and magnitude of ALAN effects on *F*_c_. The partial effect surfaces from daily models showed more regular gradients and reduced nonlinear complexity, whereas residual autocorrelation was minimal, supporting the suitability of daily models for capturing net ALAN effects on ecosystem metabolism (Extended Data Fig. 5).

**Fig. 5:**
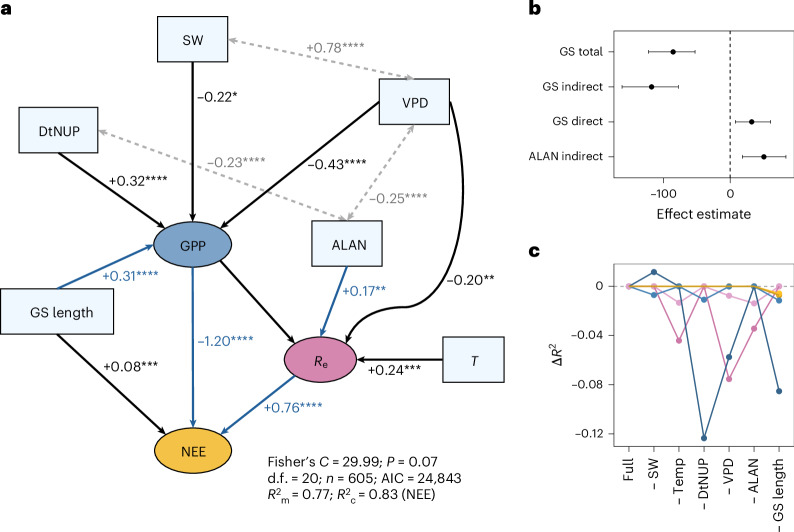
Final SEM structure for annual NEE, GPP and *R*_e_ fluxes. **a**, The hypothesized pathways linking explanatory variables to ecosystem carbon fluxes show the standardized path coefficients of the final SEM with significance levels (****P < 0.0001, ***P < 0.001, **P < 0.01, *P < 0.05, exact *P* values in Supplementary Table 10). The tests are two-sided, with no adjustments for multiple comparisons. The arrow thickness indicates the magnitude of standardized effect sizes, the black arrows and text indicate direct pathways, the double-headed grey arrows and text indicate the residual correlations and the blue arrows and text indicate the mediation pathways supported by the bootstrap analysis. The SEM was fitted to complete cases for all variables (605 site years, 84 sites). **b**, The bootstrap-derived estimates shown are means and 95% percentiles from 1,000 replicates for the direct and indirect effects of GS length and ALAN on NEE. **c**, The outputs from a leave-one-out analysis show the change in marginal (lighter-coloured symbols and lines) and conditional (darker-coloured symbols and lines) *R*² (Δ*R*²) for NEE (gold), GPP (blue) and *R*_e_ (magenta) relative to the full model after systematically removing each exogenous predictor (Supplementary Table 11).

The role of ALAN along with longer-term drivers of ecosystem metabolism was evaluated by constructing a piecewise structural equation model (SEM) integrating multiple exogenous predictors and hypothesized mediation pathways. The final SEM incorporated GS length, ALAN intensity and climatic variables including SW, VPD and *T*, along with the urban metric DtNUP (Fig. 5a). The modified Arrhenius function was not selected, reflecting how annual temporal aggregation reduces positive and negative deviations in fundamental constraints compared with short-term flux variability. The aggregated measures of ND were also not selected, with phenological drivers such as GS length more important at annual timescales (Supplementary Table 10). The mediation analysis, using nonparametric bootstrap resampling to quantify both direct and indirect effects of GS length and indirect effects of ALAN on NEE, supports the inference that the influence of ALAN on ecosystem metabolism is primarily mediated through increased *R*_e_ (Fig. 5b). The influence of GS length on NEE was significantly mediated through increased GPP (Fig. 5b). The leave-one-out sensitivity analysis of the SEM indicated that no alternative model performed better than the full model (Supplementary Table 11). Notably, the exclusion of ALAN, DtNUP, VPD or GS length led to significant declines in model performance, reflecting the importance of these predictors in explaining the interannual variation in ecosystem metabolism (Fig. 5c).

**Extended Data Fig. 6:**
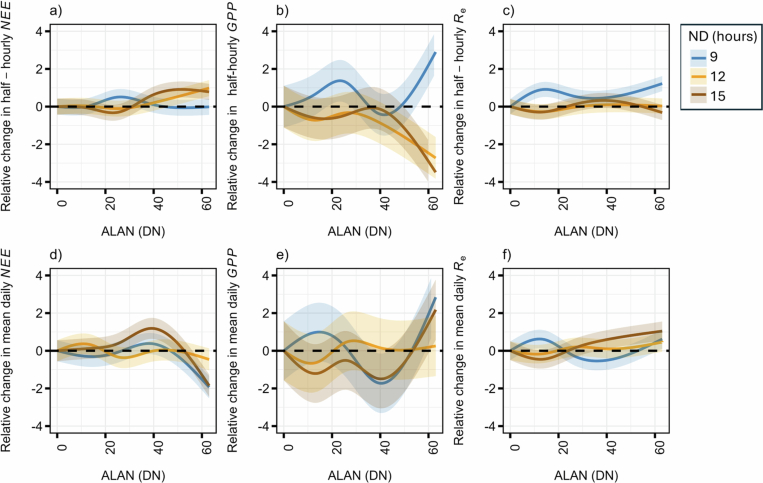
Balanced dataset GAMM predictions of half-hourly and daily carbon flux responses to ALAN and night duration. Variance-weighted GAMMs were fitted to the balanced ALAN intensity dataset, comprising an equal number of sites (n = 4 each in low, medium, and high ALAN intensity categories per continent). Panels (**a–c**) show predicted relative changes in half-hourly (**a-c**) and mean daily (**d-f**) carbon fluxes for *NEE* (**a, d**), *GPP* (**b, e**), *R*_e_ (**c, f**) as a function of ALAN (DN) intensity. Coloured lines represent night duration (ND) categories of 9, 12, and 15 h, with shaded ribbons representing mean predictions ±95% confidence intervals. The horizontal dashed line denotes zero relative change.

**Extended Data Fig. 7:**
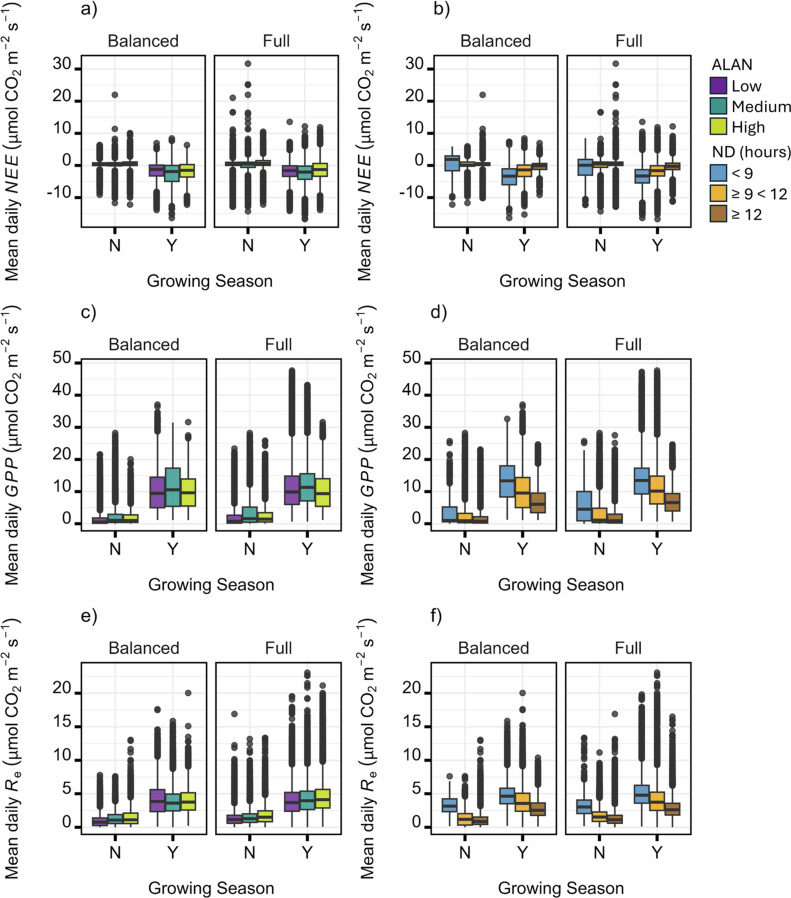
Distributions of mean daily carbon fluxes in the balanced versus full datasets. The balanced dataset represents equal distribution of sites in ALAN intensity groups (n = 34,351 across 24 sites) compared to the full dataset (n = 231,598 across 86 sites). Boxplots for (**a, b**) *NEE*, (**c, d**) *GPP*, and (**e, f**) *R*_e_ are stratified by growing season (N: non-growing season, Y: growing season) and grouped according to (left: **a, c, e**) ALAN intensities or (right: **b, d, f**) Night Duration (ND). Boxes represent interquartile ranges (IQR), horizontal lines denote medians, whiskers extend to 1.5 × IQR, and points indicate outliers.

**Extended Data Fig. 8:**
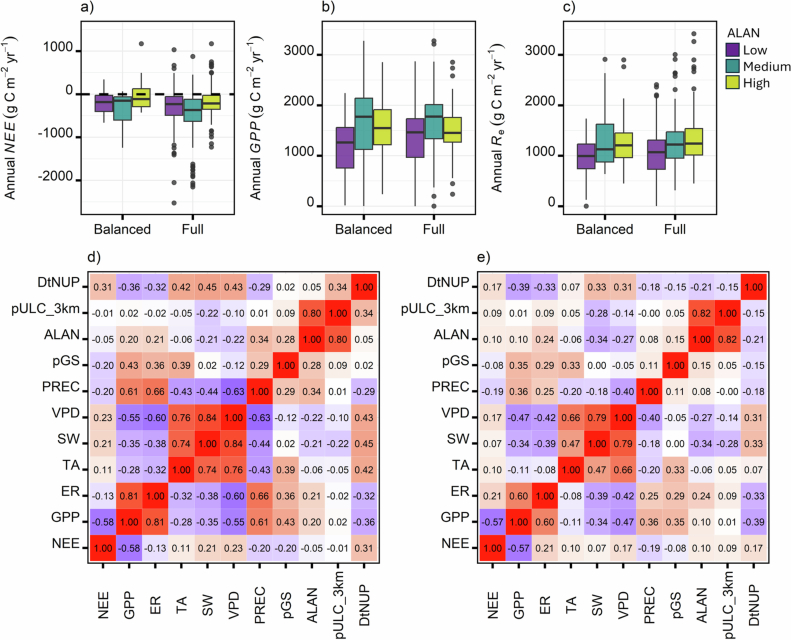
Comparison of annual carbon fluxes and correlations with environmental variables in full and balanced datasets. Panels show annual: **a**) *NEE*, **b**) *GPP* and **c**) *R*_e_ grouped by ALAN intensity. Boxes (**a-c**) represent interquartile ranges (IQR), horizontal lines indicate medians, whiskers extend to 1.5 × IQR, and points denote outliers for the balanced dataset (with equal representation of sites across ALAN intensity groups, n = 120) and the full dataset (n = 605). Correlation matrices between annual environmental and urban variables with carbon fluxes are shown for (**d**) the balanced dataset and (**e**) the full dataset. Pearson correlation coefficients are shown, with colour intensity indicating the strength (lighter: weaker, darker: stronger correlation) and direction (red: positive, blue: negative, white: none) of correlations.

**Extended Data Fig. 9:**
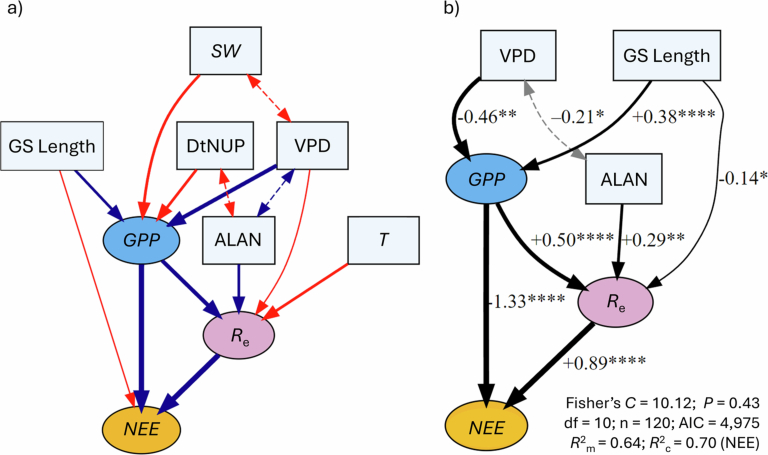
Sensitivity analysis of annual structural equation models fitted to the balanced dataset. Panel (**a**) illustrates the original structural equation model (SEM) structure (Fig. 5, fitted to the full dataset). The balanced dataset yielded an acceptable SEM fit to the original model in (**a**) Fisher’s *C* = 25.05, *P* = 0.199, df = 20, n = 120, AIC = 4,939, *R*^2^_m_ = 0.64, *R*^2^_c_ = 0.71 (*NEE*) but interactions between *GPP* ~ *SW* + DtNUP, *R*_e_ ~ *T* + VPD, and *NEE* ~ GS Length were no longer significant (*p* > 0.05, red lines in **a**). Retained pathways (blue lines in **a**) were fitted to the balanced dataset alongside *R*_e_ ~ GS Length, which was identified as a missing pathway in the balanced SEM. Standardized path coefficients are shown for the balanced SEM in (**b**), adjacent to arrows with line thickness indicating the relative strength of relationships. Significance levels for path coefficients (solid black arrows in **b**), *p* < 0.0001: ****, *p* < 0.001 ***, *p* < 0.01 **, *p* < 0.05 *) are based on two-sided tests, with no multiple comparison adjustments. The refitted SEM to the balanced dataset (**b**) had a better global fit (SEM diagnostics shown in plot) than (**a**) but yielded a higher AIC due to the original SEM in (**a**) having better explanatory power for *R*_e_ (a: *R*^2^_m_ = 0.51; b: *R*^2^_m_ = 0.38).

To ensure our data analysis was robust to site bias across ALAN intensities (17, 22 and 47 sites at high, medium and low ALAN intensities, respectively, and 34 sites in North America and 52 sites in Europe), we repeated all GAMM and SEM analyses using a balanced dataset with an equal representation of low, medium and high ALAN intensity sites per continent (Extended Data Figs. 6–9). The models fitted to the balanced dataset showed consistently significant nonlinear interactions between ALAN and ND across temporal scales (Extended Data Fig. 6), and the annual SEM retained the core structure of ALAN indirectly influencing NEE through increased *R*_e_ (Extended Data Fig. 9). Whereas several weaker interactions (for example *R*_e_ ~ *T* and NEE ~ GS length) were no longer significant owing to reduced sample size, the SEM retained dominant pathways, and the overall explanatory power was comparable (*R*^2^_m_ = 0.64, *R*^2^_c_ = 0.70 for NEE). Notably, the standardized coefficients strengthened between ALAN ~ *R*_e_ and *R*_e_ ~ NEE when the SEM was fitted to the balanced dataset. Our observed ALAN effects on ecosystem metabolism are therefore robust to spatial imbalances in ALAN intensity across FLUXNET2015 site distribution.

Our study provides cross-continental evidence of ALAN’s influence on ecosystem metabolism across timescales. We demonstrate that ALAN consistently modifies the relationship between *F*_c_ and their fundamental energetic constraints (Figs. 2–4). The *R*_e_ response to fundamental constraints was particularly sensitive to ALAN intensity at short (half-hourly and daily) timescales (Fig. 2 and Supplementary Tables 2 and 6). Alongside *R*_e_, GPP and NEE exhibited significant nonlinear interactions between ND and ALAN intensity, revealing the importance of ALAN magnitude and timing in modulating ecosystem metabolism across scales (Figs. 3 and 4). At annual timescales, the influence of ALAN on NEE was primarily mediated through increased *R*_e_ rather than the direct suppression of GPP (Fig. 5). Taken together, our findings demonstrate the role of ALAN as a pervasive stressor capable of disrupting carbon balance across spatial and temporal scales.

The nonlinear influence of ALAN on ecosystem metabolism was strongly modulated by diel cycles and seasonality, demonstrating the importance of phenological dynamics^34^ and biogeochemical feedbacks in shaping long-term carbon balance^35^. The temporal aggregation led to notable shifts in the strength and direction of ALAN effects on *R*_e_, whereas GPP and NEE displayed more consistent nonlinear responses to ND across timescales (Figs. 3 and 4). At the half-hourly resolution, short nights showed the strongest ALAN-induced increases in *R*_e_ (Fig. 3h,i), reflecting immediate physiological and microbial responses such as prolonged stomatal opening^36^, sustained leaf dark respiration^37^ and elevated microbial decomposition under disrupted circadian regulation^38^. By contrast, daily mean nighttime *R*_e_ estimates indicated larger ALAN-related increases during longer nights (Fig. 4f), demonstrating how aggregation dampens short-term variability while revealing broader shifts in *R*_e_ across longer nights. GPP exhibited consistent positive or nonlinear ALAN effects across timescales (Figs. 3 and 4), probably driven by nocturnal illumination extending photosynthetic activity at medium ALAN intensities^39,40^. Temporal scale and ND thus collectively shape ALAN’s ecological impact, whereas diel averaging can obscure short-lived physiological responses while reflecting cumulative nighttime effects.

*R*_e_ exhibited greater sensitivity to ALAN than GPP across timescales (Figs. 2 and 3 and Supplementary Tables 2–11), and the SEM confirmed that ALAN primarily influences NEE indirectly via increased *R*_e_ at annual timescales (Fig. 5). The destabilizing effect of ALAN on production-respiration coupling will arise from shifts in multiple autotrophic and heterotrophic processes controlling carbon allocation and use efficiency^17,38^. The greater *R*_e_ sensitivity may reflect a higher capacity of autotrophs to acclimate to ALAN through conservative growth strategies such as increased shoot-to-root ratios^20,41^. In ecosystems dominated by C_3_ plants, for instance, prolonged ALAN exposure can disrupt circadian regulation and prolong stomatal opening, reducing carbon uptake efficiency, increasing mortality and senescence, and leading to reduced GPP over time^42,43^. Such trophic mismatches and shifts in carbon allocation are likely to accumulate across levels of biological organization, space and time^44,45^, leading to progressive declines in NEE in illuminated ecosystems.

The ecological impacts of ALAN have primarily been examined at local scales^6,17^, but landscape-scale factors will confound or amplify these localized effects^2^. Urban proximity influenced GPP in our analysis, whereas ALAN directly influenced *R*_e_ (Fig. 5), suggesting distinct pathways through which nighttime lighting and urban characteristics modify ecosystem metabolism. Balancing sites across low, medium and high ALAN intensities further indicates potentially stronger mediating effects of ALAN on NEE via *R*_e_ (Extended Data Fig. 9). Despite the pervasive nature of light pollution, ALAN remains overlooked in ESM carbon–climate projections that otherwise account for climate and land use changes. Current observational data, however, do not enable the disentangling of the contribution of ALAN relative to sunlight in shaping *F*_c_, and future targeted experimental studies will be needed to resolve these relationships.

Global eddy covariance networks such as FLUXNET are vital for monitoring ecosystem metabolism across diverse climates and land use types, but they are typically biased towards temperate regions, seminatural landscapes and dark skies (Extended Data Fig. 1). Urban flux towers are particularly scarce, and although networks such as Urban PLUMBER have been established, they do not measure *F*_c_ (ref. ^46^). Similarly, available nighttime light satellite products used here (Visible Infrared Imaging Radiometer Suite (VIIRS) and Defence Meteorological Satellite Programme (DMSP)) are coarse in spatial resolution, are insensitive to blue light emitted by white light-emitting diode (LED) lighting^47^ and cannot fully capture local heterogeneity in ALAN exposure at flux tower sites.

Enhanced satellite sensors with improved spectral and spatial resolution would advance ALAN monitoring^2^, but ground-based measurements are also needed to capture how cloud cover exacerbates or reduces skyglow (brightening of the night sky) in high or low ALAN intensity areas, respectively^48^. The coordinated expansion of eddy covariance flux tower networks along with complementary measurements, such as chamber-based respiration estimates and isotopic tracers, will be critical to disentangle the mechanisms by which ALAN alters ecosystem metabolism. Expanding ecosystem-level *F*_c_ measurements into urbanized, tropical, arid and high-latitude regions is vital to evaluate the global relevance of ALAN impacts on carbon cycling. While monitoring is essential, mitigation is also readily achievable.

Artificial light is ubiquitous and often beneficial, but the negative ecological effects of light pollution can be reduced while balancing societal benefits. Retrofitting LED lighting can reduce light pollution^5^, but it often results in over-illumination due to their higher efficiency^49^. Given that lighting accounts for 20% of global electricity consumption and 6% of CO_2_ emissions^50^ and can exacerbate degraded air quality^51,52^, mitigation interventions such as directional, dimmable and adaptive lighting designs^2^ offer wider cobenefits. Unlike climate and land use change, the effects of light pollution could be mitigated overnight^53^. Our study demonstrates the pervasive influence of light pollution on ecosystem metabolism across scales and highlights the urgent need to integrate ALAN into global change research, assessments of carbon–climate feedbacks and mitigation strategies. Developing a higher resolution understanding of species, community and ecosystem sensitivity to ALAN will be central to designing interventions that both safeguard biodiversity and preserve the land carbon sink.

## Methods

We combined satellite-derived ALAN intensity metrics with eddy covariance flux measurements from 86 FLUXNET2015 sites in North America and Europe. The analyses were conducted at half-hourly, daily and annual timescales to capture short-term physiological responses, aggregated diel patterns and long-term ecosystem dynamics. All *F*_c_ (NEE, GPP and *R*_e_) were first evaluated against their fundamental energetic constraints of *T* and SW according to the modified Arrhenius function (equation (1)) using LMMs. To explore nonlinear interactions, we then applied GAMMs which allow the flexible estimation of smooth terms. At annual timescales, we used piecewise SEM to account for collinearity among multiple drivers and to partition direct and indirect effects of ALAN, GS length and climatic variables on *F*_c_. This hierarchical modelling framework enabled the consistent evaluation of ALAN effects across temporal scales.

### Global harmonized nighttime light dataset

The Day/Night Band of the VIIRS is the only satellite radiometer currently acquiring imagery of the Earth at night, providing single-band nightscapes at a resolution of 750 m since 2012 across the globe. Prior global nightscapes were monitored by DMSP–Operational Linescan System (OLS) at a resolution of 1 km between 1992 and 2013. A harmonized global nighttime light dataset, developed by Li et al.^32^, provides a consistent annual time-series of overall luminance between 1992 and 2018 at ~1 km resolution through the intercalibration of DMSP-like DN values. The DNs for all FLUXNET2015 sites (see below) and site years were derived from the harmonized dataset of Li et al.^32^. The low DN sites (DN <10) were cross-checked in Google Earth for each site year to verify low DN values reflected remote locations by noting distances to the nearest built-up area in QGIS (version 3.30.3). The satellite-derived DN values from DMSP–Operational Linescan System and VIIRS represent relative radiance indices and cannot be directly converted into absolute illuminance units such as lux, as they do not capture spectral composition or ground-level variability^54^. The ALAN categories used here should thus be interpreted as relative exposure gradients rather than specific ecological thresholds.

### FLUXNET dataset and site selection

FLUXNET is a global network of micrometeorological sites providing eddy covariance CO_2_ exchange observations between terrestrial ecosystems and the atmosphere^30^. The FLUXNET2015 dataset used in this study includes measurements from 210 eddy covariance flux towers across the globe^30^. A total of five tier-2 sites and two arctic sites outside the latitudinal range of the global nighttime light dataset (latitude >75° N) were excluded. Originally, DNs from the harmonized nighttime light dataset were extracted for 203 FLUXNET2015 sites from 1992 to 2014 (1,474 site years) (Extended Data Fig. 1). The 203 sites were composed of 1,116 low (DN <10), 243 medium (DN ≥ 10 < 30) and 115 high (DN >30) ALAN intensity site years. Only one high ALAN intensity FLUXNET2015 site (JP-SMF) was located outside of North America or Europe, with no replication of low ALAN intensity sites in a 2° latitudinal or longitudinal range. The site selection was therefore restricted to North America and Europe to reduce noise from additional climatic and ecosystem properties at low ALAN intensity FLUXNET2015 sites globally. Within both North America and Europe, the latitudinal and longitudinal ranges of selected FLUXNET2015 sites were based on the presence of medium or high ALAN intensity sites at 2° intervals (Fig. 1 and Supplementary Table 1).

Disentangling respiration and photosynthesis fluxes during the day is complex and relies on modelling techniques with high uncertainty, particularly under low turbulence or during transitional periods around dawn and dusk. The FLUXNET2015 dataset undergoes processing to check data quality, filter low turbulence periods and CO_2_ flux partitioning into respiration and photosynthesis using established methods^30^. The measurements were compiled from the FLUXNET2015 dataset^55^, which in this study includes non-gap-filled half-hourly and annual air temperature (TA_F), incoming shortwave (SW_IN_F), NEE (NEE_VUT), nighttime *R*_e_ (RECO_NT) and daytime GPP (GPP_DT) measurements for 86 sites across 623 site years. Along with the use of nighttime *R*_e_ and daytime GPP, the half-hourly data were filtered for *R*_e_ by selecting timepoints with GPP <0.001 µmol CO_2_ m^−2^ s^−1^ and outgoing SW greater than incoming SW and vice versa for daytime GPP.

Additional environmental and urban variables were derived to check for confounding effects, including half-hourly and annual VPD (VPD_F) and P (P_F) from FLUXNET2015. Urban metrics pULC_3km, pULC_10km and DtNUP were calculated by quantifying the proportion of land cover classified as urban within 3- and 10-km buffers around each site, using the ESA CCI Land Cover dataset, and computing the Euclidean distance (km) from the site centroid to the nearest urban polygon in the Copernicus Urban Centre Database^56^. To account for latitudinal and climatic variation in phenology across the 86 sites, GS was classified using site-specific 25th percentiles of daily GPP per site year. GPP measurements above the 25th percentile threshold was classified as occurring within the GS, and other observations were classified as non-GS. To avoid classifying transient periods of activity as part of the GS, we implemented a hybrid phenological rule requiring ≥5 consecutive candidate days for GS initiation, and ≥5 consecutive non-GS days to mark the GS finish. To estimate the daily duration of night at each study site, we calculated the time between astronomical sunset and sunrise (UTC) using site-specific latitude, longitude and observation dates using the suncalc package. The ND was calculated as the time in hours elapsed between sunset on a given day and sunrise on the following day.

### Model analysis

All model analyses were conducted in R statistical software (version 4.2.2)^57^. The null models (equation (1)) describe the relationship between *F*_c_ (NEE, GPP and *R*_e_) and their fundamental constraints (SW and *T*) according to a modified Arrhenius function^12^. Unlike alternative functions such as those used in metabolic ecology^26^, the modified Arrhenius function enables the exploration of NEE, GPP and *R*_e_ according to a single measure of shared fundamental constraints and analysis of untransformed *F*_c_ measurements. The null models for NEE, GPP and *R*_e_ according to equation (1) were fitted to half-hourly, daily and annual FLUXNET2015 measurements, with LMMs and GAMMs applied to half-hourly and daily measurements and an SEM developed for annual timescales.

### LMMs

First, LMMs were incrementally tested for each carbon flux and explanatory variable: continent (North America and Europe), climate (boreal, temperate and Mediterranean), International Geosphere–Biosphere Programme land use classifications (CRO, CSH, DBF, EBF, ENF, GRA, MF and WET), GS (Y and N), ND (hours), month, hour of the day, VPD, P, pULC_3km, pULC_10km, DtNUP and ALAN. FLUXNET2015 site (*n* = 86) and latitude (*n* = 80) were included as random effects to account for spatial clustering. The model selection thresholds (ΔAIC_df_ <−5 and Δ*R*^2^_mdf_ >0.01) ensured that any increase in explanatory power was proportionate to model complexity and prevented LMM overfitting by only relying on ΔAIC selection criteria. The bootstrapped 95% confidence intervals for LMM marginal and conditional *R*^2^ fits were computed by resampling model residuals using 500 semiparametric bootstrap replicates. For the half-hourly datasets (4–10 million observations) we used random 10% subsamples to provide reliable estimates while avoiding computational limitations inherent in very large datasets.

### GAMMs

GAMMs were used to explore nonlinear relationships and interactions between variables, with initial GAMMs including variables identified as potentially important in the LMMs for the temporal resolution (half-hourly and daily) under consideration. The smooth terms were specified for continuous explanatory variables, and the categorical variables were treated as parametric effects. The tensor product interactions and stratification of categorical variables were also tested where ecologically feasible, and FLUXNET2015 site and latitude were included as random smooth terms. All final GAMMs identified latitude as a redundant random effect, defined statistically as a lack of improvement in model fit and a concurvity value of 1, indicating complete collinearity with other smooth terms. We use a backward selection approach to sequentially simplify the initial models, with GAMM selection based on a combination of penalized likelihood (fREML), *R*^2^_adj_, deviance explained (%) and approximate significance of smooth terms. We did not apply *R*^2^_adj_ per df thresholds as with LMMs, as GAMMs inherently penalize smooth term complexity during estimation to optimize effective df.

To address the risk of overfitting and concurvity (collinearity between smooth terms), we evaluated GAMM diagnostics and smooth terms exhibiting high concurvity values (>0.8) were identified as potentially redundant. Preference during model selection was given to simpler models that retained comparable, although usually lower, explanatory power while reducing concurvity. Additional explanatory variables were then reintroduced to the final backward selected GAMMs to compare model performance. Finally, heteroscedasticity (non-constant residual variance) in the final GAMMs was accounted for by comparing the final selected GAMMs to variance-weighted GAMMs, which provide lower weight to observations associated with higher residual variance. Model performance and smooth term significance were compared between the unweighted and weighted GAMMs to ensure robust evidence for the selected explanatory variables. For all GAMMs, residual autocorrelation was evaluated using partial autocorrelation functions.

### SEM

To investigate the relationships between environmental drivers and annual NEE, GPP and *R*_e_, we developed a piecewise SEM comprising three linked LMMs, including NEE ~ GPP + *R*_e_, GPP ~ SW and *R*_e_ ~ GPP + *T* and testing additional exogenous predictors (GS length, mean ND, VPD, P, ALAN, pULC_3km, pULC_10km and DtNUP). The annual dataset included 605 site years across 84 sites after excluding site years with missing variables. Both LMMs and GAMMs were evaluated for component models. Given the relatively small sample size, GAMMs presented a higher risk of overfitting and unstable smooth functions at the annual timescale. The annual aggregation of *F*_c_ measurements also inherently smoothed diel and seasonal nonlinearities observed in half-hourly and daily measurements. Exploratory diagnostics further indicated that annual relationships were approximately linear, supporting the use of LMMs as a parsimonious framework capable of accounting for site-level random intercepts while estimating fixed effects on annual *F*_c_. The SEMs included residual covariance terms among exogenous predictors to account for collinearity, and model fit between SEM’s was evaluated through Fisher’s *C* and *P*, AIC and df and marginal and conditional *R*^2^ for NEE, GPP and *R*_e_.

The mediation pathways in the final SEM quantified the indirect effect of GS length on NEE via GPP and the indirect effect of ALAN on NEE via *R*_e_. The uncertainty in direct and indirect effects was estimated through nonparametric bootstrap resampling (1,000 iterations). In each iteration, the three component LMMs were refitted on a bootstrap-resampled dataset with replacement, and indirect effects were calculated as the product of relevant path coefficients. Percentile bootstrap confidence intervals (95%) were derived for each estimated effect and considered significant if they did not overlap zero. The relative importance of each exogenous predictor in the final SEM was inferred through a leave-one-out sensitivity analysis, in which each variable was removed in turn and the reduced SEM fit compared, rather than absolute effect sizes. The model fit for each alternative SEM specification fitted to the same dataset was compared with the full model, with higher model sensitivity indicated by a significantly poorer fit (*P* < 0.05), higher AIC, or reduced explanatory power (*R*^2^_m_ and *R*^2^_c_ for NEE, GPP and *R*_e_) relative to the final selected SEM.

### Sensitivity analysis

The sensitivity of models to the composition of FLUXNET2015 sites was evaluated by generating a balanced, stratified subset of the full dataset with equal representation of low, medium and high ALAN intensity sites across both continents at half-hourly, daily and annual timescales. A random sample of sites equal to the stratum with the fewest available sites (high ALAN sites in North America, *n* = 4) was selected without replacement from each stratum (4 × 2 continents × 3 ALAN intensity groups = 24 sites in the balanced dataset). The half-hourly and daily GAMMs and the annual SEM were refitted to the balanced dataset using the same model specification in the main analysis.

### Reporting summary

Further information on research design is available in the Nature Portfolio Reporting Summary linked to this article.

## Online content

Any methods, additional references, Nature Portfolio reporting summaries, source data, extended data, supplementary information, acknowledgements, peer review information; details of author contributions and competing interests; and statements of data and code availability are available at 10.1038/s41558-025-02481-0.

## Supplementary information

Supplementary Information Supplementary Tables 1–11.

Reporting summary

## Data Availability

The FLUXNET2015 data analysed in this study are available at https://fluxnet.fluxdata.org/data/fluxnet2015-dataset/ (ref. ^55^) and are subject to the FLUXNET data policy (https://fluxnet.org/data/data-policy). As the redistribution of raw half-hourly flux data is not permitted, we provide only derived products, including daily and annual summaries, processed variables and model outputs, which are available under a CC-BY 4.0 license via Figshare at 10.6084/m9.figshare.29958455 (ref. ^58^). The ALAN metrics used here are available at 10.3390/rs9060637 (ref. ^32^). Summaries for each FLUXNET site are also provided in Supplementary Table 1.

## Code availability

The R code used for data processing and analysis in this study is available via Figshare at 10.6084/m9.figshare.29958455 (ref. ^58^).
