# Supplementary material for: Widespread influence of artificial light at night on ecosystem metabolism
Source: Nat Clim Chang. 2025 Nov 12;15(12):1371–7. doi: 10.1038/s41558-025-02481-0 (PMC12672368; doi:10.1038/s41558-025-02481-0)
Supplement: Supplementary file 1 — Supplementary Tables 1–11. [file 41558_2025_2481_MOESM1_ESM.pdf]

nature climate change

Supplementary information https://doi.org/10.1038/s41558-025-02481-0

Widespread influence of artificial light at night on ecosystem metabolism

In the format provided by the authors and unedited

Supplementary Table 1. Summary of FLUXNET2015 sites included in the analysis conducted here (see Methods for site selection). Cont. indicates Continent (E: Europe, NA: North America), IGBP indicates land use classifications, ALAN (DN) is mean ±standard error digital number for each site, and NEE is mean ±standard error annual net ecosystem exchange for each site, across site years (N).

Site	Lat (°)	Lon (°)	Cont.	IGBP	ALAN (DN)	Annual NEE (g C m-2 yr-1)	N
AT-NEU	47.12	11.32	E	GRA	33 ±1.49	374 ±6.50	10
BE-BRA	51.31	4.52	E	MF	54 ±0.94	21.50 ±1.83	14
BE-LON	50.55	4.75	E	CRO	23 ±1.11	-337 ±2.90	9
BE-VIE	50.30	6.00	E	MF	7 ±0.30	-503 ±2.38	17
CA-TP2	42.77	-80.46	NA	ENF	10 ±0.70	-879 ±10.77	4
CA-TP3	42.71	-80.35	NA	ENF	8 ±0.46	-486 ±2.81	10
CA-TP4	42.71	-80.36	NA	ENF	7 ±0.43	-85.90 ±4.00	11
CA-TPD	42.64	-80.56	NA	DBF	4 ±1.86	-299 ±4.35	2
CH-CHA	47.21	8.41	E	GRA	26 ±2.30	-14.75 ±18.23	8
CH-DAV	46.82	9.86	E	ENF	30 ±1.60	-181 ±23.47	16
CH-FRU	47.12	8.54	E	GRA	13 ±0.87	-350 ±7.68	8
CH-LAE	47.48	8.37	E	MF	34 ±2.98	-648 ±28.20	9
CH-OE1	47.29	7.73	E	GRA	32 ±1.79	-316 ±1.57	6
CH-OE2	47.29	7.73	E	CRO	35 ±1.66	-17.98 ±3.15	9
CZ-WET	49.02	14.77	E	WET	27 ±2.02	-57.70 ±2.43	8
DE-AKM	53.87	13.68	E	WET	36 ±3.12	13.44±2.84	5
DE-GEB	51.10	10.91	E	CRO	10 ±0.90	-212 ±1.78	12
DE-GRI	50.95	13.51	E	GRA	8 ±0.49	-169 ±4.03	9
DE-HAI	51.08	10.45	E	DBF	0	-566 ±3.63	12
DE-KLI	50.89	13.52	E	CRO	10 ±1.41	-256 ±1.78	9
DE-LKB	49.10	13.30	E	ENF	0	278 ±3.10	4
DE-LNF	51.33	10.37	E	DBF	7 ±0.49	-602 ±4.21	8
DE-OBE	50.79	13.72	E	ENF	19 ±2.39	-375 ±1.88	6
DE-RUR	50.62	6.30	E	GRA	21 ±4.55	-147 ±1.44	3
DE-RUS	50.87	6.45	E	CRO	37 ±3.57	-598 ±1.08	3
DE-SEH	50.87	6.45	E	CRO	38 ±3.28	-672 ±4.75	4
DE-SFN	47.81	11.33	E	WET	8 ±1.15	-343 ±3.88	2
DE-SPW	51.89	14.03	E	WET	1 ±1.00	-543 ±4.03	4
DE-THA	50.96	13.57	E	ENF	13 ±0.89	-629 ±1.86	17
DE-ZRK	53.88	12.89	E	WET	6 ±0.00	44.61 ±8.16	1
DK-ENG	55.69	12.19	E	GRA	15 ±0.50	-188 ±2.20	3
DK-FOU	56.48	9.59	E	CRO	7	-22.86 ±1.05	1
DK-SOR	55.49	11.64	E	DBF	6 ±0.47	-208 ±2.36	17
FR-FON	48.48	2.78	E	DBF	17 ±0.81		8
FR-GRI	48.84	1.95	E	CRO	54 ±1.35	-534 ±2.37	9
FR-LBR	44.72	-0.77	E	ENF	12 ±1.27	-398 ±4.62	12
FR-PUE	43.74	3.60	E	EBF	4 ±0.69	-228 ±3.46	13
IT-BCI	40.52	14.96	E	CRO	17 ±0.79	-218 ±3.36	9
IT-CA1	42.38	12.03	E	DBF	10 ±0.87	-38 1 ±3.28	3
IT-CA3	42.38	12.02	E	DBF	9 ±1.03	-463 ±3.42	3
IT-COL	41.85	13.59	E	DBF	3 ±0.62	-835 ±6.44	17
IT-CP2	41.70	12.36	E	EBF	37 ±9.21	-697 ±3.27	2
IT-CPZ	41.71	12.38	E	EBF	19 ±1.09	-554 ±2.85	9

Site	Lat (°)	Lon (°)	Cont.	IGBP	ALAN (DN)	Annual NEE (g C m-2 yr-1)	N
IT-ISP	45.81	8.63	E	DBF	45 ±4.00	-84.27 ±18.12	1
IT-LA2	45.95	11.29	E	ENF	12 ±0.58	-1375 ±17.02	3
IT-LAV	45.96	11.28	E	ENF	15 ±0.84	-1841 ±1.83	10
IT-MBO	46.01	11.05	E	GRA	9 ±0.37	-45.29 ±1.63	9
IT-NOE	40.61	8.15	E	CSH	7 ±0.41	-214 ±6.90	9
IT-PT1	45.20	9.06	E	DBF	13 ±0.33	-540 ±5.16	3
IT-REN	46.59	11.43	E	ENF	2 ±0.63	-515 ±7.87	10
IT-RO1	42.41	11.93	E	DBF	3 ±0.82	-254 ±5.40	8
IT-RO2	42.39	11.92	E	DBF	1 ±0.56	-678 ±5.56	9
IT-SR2	43.73	10.29	E	ENF	8 ± 0.00	-361 ±3.92	1
IT-TOR	45.84	7.58	E	GRA	10 ±1.13	-143 ±7.48	6
NL-HOR	52.24	5.07	E	GRA	31 ±1.62	-277 ±2.00	7
NL-LOO	52.17	5.74	E	ENF	10 ±0.38	-409 ±3.20	17
US-AR1	36.43	-99.42	NA	GRA	55 ±1.70	105 ±2.87	4
US-AR2	36.64	-99.60	NA	GRA	0	34.79 ±1.99	4
US-ARB	35.55	-98.04	NA	GRA	13 ±1.50	-30.42 ±2.53	1
US-ARM	36.61	-97.49	NA	CRO	1 ±0.60	-42.68 ±3.72	9
US-BLO	38.90	-120.63	NA	ENF	0	-435 ±26.32	10
US-COP	38.09	-109.39	NA	GRA	0	-36.94 ±0.38	6
US-CRT	41.63	-83.35	NA	CRO	24 ±0.33	-91.52 ±2.30	2
US-GBT	41.37	-106.24	NA	ENF	0		3
US-GLE	41.37	-106.24	NA	ENF	1 ±0.55	79.73 ±13.55	8
US-GOO	34.25	-89.87	NA	GRA	7 ±0.66	-266 ±3.37	4
US-HA1	42.54	-72.17	NA	DBF	7 ±0.23	-205 ±10.09	20
US-IB2	41.84	-88.24	NA	GRA	56 ±0.37	-164 ±1.74	7
US-KS1	28.46	-80.67	NA	ENF	13	-289 ±7.07	1
US-KS2	28.61	-80.67	NA	CSH	30 ±2.48	-295 ±2.02	3
US-LIN	36.36	-119.09	NA	CRO	7 ±0.50	-310 ±7.13	2
US-LWW	34.96	-97.98	NA	GRA	8 ±0.50	250 ±0.50	2
US-MMS	39.32	-86.41	NA	DBF	2 ±0.66	-428 ±2.42	14
US-MYB	38.05	-121.77	NA	WET	12 ±1.24	-71.59 ±5.94	3
US-NE1	41.17	-96.48	NA	CRO	8 ±0.25	-265 ±4.11	11
US-NE2	41.16	-96.47	NA	CRO	8 ±0.29	-242 ±3.46	11
US-NE3	41.18	-96.44	NA	CRO	8 ±0.35	-171 ±5.62	11
US-NR1	40.03	-105.55	NA	ENF	0	-148 ±1.00	15
US-OHO	41.55	-83.84	NA	DBF	18 ±0.86	-845 ±4.95	8
US-ORV	40.02	-83.02	NA	WET	63	-217 ±7.21	1
US-SRG	31.79	-110.83	NA	GRA	0	-11.33 ±2.18	6
US-TW2	38.10	-121.64	NA	CRO	6 ±0.50	814 ±6.57	1
US-TW3	38.12	-121.65	NA	CRO	7 ±0.50	-732 ±10.32	1
US-TWT	38.11	-121.65	NA	CRO	6 ±0.43	107 ±7.70	5
US-WKG	31.74	-109.94	NA	GRA	0	-9.08 ±2.01	9
US-WPT	41.46	-83.00	NA	WET	4 ±2.19	203 ±4.54	2

Supplementary Table 2. Null linear mixed effect models (LMMs) fitted to half-hourly ecosystem carbon flux (*NEE*, *GPP* and R_e) observations with FLUXNET2015 site (n = 86) and latitude (n = 80) set as random effects, showing model goodness of fits with additional selected terms (growing season, continent, climate, IGBP land use classification, night duration, hour of the day, vapor pressure deficit (VPD), precipitation, ALAN, distance to nearest urban polygon (DtNUP) and proportion urban land cover in 3 and 10 km buffers of each site tested). Terms that were not selected based on model fit and parsimony conditions (ΔAIC_{df} < -5 and ΔR^2_{mdf} > 0.01) are not shown. Final selected model results are presented in Supplementary Table 3.

**Fixed effects**	**df**	R^2_m	R^2_c	ΔAIC_{df}	ΔR^2_{mdf}
**Net ecosystem exchange (*NEE*) n = 10,069,905**					
NEE ~ $T^4\sigma\ e^{\frac{-SW}{\sigma T^4}}$ (null)	5	0.462	0.492	0	0
+ Growing Season	6	0.498	0.522	-714,871	+0.036
**Gross primary production (*GPP*) n = 5,340,294**					
GPP ~ $T^4\sigma\ e^{\frac{-SW}{\sigma T^4}}$ (null)	5	0.305	0.426	0	0
+ Growing Season	6	0.498	0.589	-1,948,888	+0.193
+ Night Duration	7	0.508	0.602	- 175,103	+0.010
+ DtNUP	8	0.531	0.597	-18.80	+0.023
**Ecosystem respiration (R_e) n = 4,181,247**					
R_e ~ $T^4\sigma\ e^{\frac{-SW}{\sigma T^4}}$ (null)	5	0.415	0.679	0	0
+ Growing Season	6	0.459	0.692	-460,353	+0.044
+ Night Duration	7	0.468	0.700	-242,720	+0.010
+ VPD	8	0.487	0.701	-94,407	+0.019
+ ALAN	9	0.499	0.713	-16,842	+0.012

Supplementary Table 3. Final LMMs with selected terms as presented in Supplementary Table 2. All models were for half-hourly carbon flux measurements (μmol CO_2 m^{-2} s^{-1}), with all *NEE*, daytime *GPP* and nighttime R_e measurements (see Methods). FLUXNET2015 site (n = 86) and latitude (n = 80) were set as random effects in all models. Model fits are shown for each final model, including bootstrapped 95% confidence intervals from 500 semiparametric bootstrap replicates.

Net ecosystem exchange: *NEE* ~ $T^4\sigma\ e^{\frac{-SW}{\sigma T^4}}$ + GS + (1|FLUXNET2015 site) + (1|Latitude)

Random effects	Variance	SD	Fixed effects	Estimate ±SE	T value
FLUXNET2015 site	0.582	0.763	Intercept	-11.11±0.114	-98.03
Latitude (°)	0.479	0.692	$T^4\sigma\ e^{-SW/\sigma T4}$	0.042 ±1.35×10^{-5}	3101.66
Residual	21.12	4.59	Growing Season	-2.58 ±2.99×10^{-3}	-860.74

AIC = 59,295,065; df = 6; R^2_m = 0.498; R^2_c = 0.522, n = 10,069,905; Bootstrapped R^2_m = 0.497 (95% CI: 0.496-0.500)

Gross primary production: *GPP* ~ $T^4\sigma\ e^{\frac{-SW}{\sigma T^4}}$ + GS + ND + DtNUP + (1|FLUXNET2015 site) + (1|Latitude)

Random effects	Variance	SD	Fixed effects	Estimate ±SE	T value
FLUXNET2015 site	0.789	0.889	Intercept	20.53 ±0.034	59.69
Latitude (°)	4.14	2.04	$T^4\sigma\ e^{-SW/\sigma T4}$	-0.043 ±2.28 $\times10^{-5}$	-1863.38
Residual	30.09	5.49	Growing Season	5.90 ±6.67×10^{-3}	884.06
			Night Duration	-0.55 ±1.31×10^{-3}	-421.93
			DtNUP	-0.030 ±4.95 $\times10^{-3}$	-5.95

AIC = 33,335,551; df = 8; R^2_m = 0.531; R^2_c = 0.597, n = 5,340,294; Bootstrapped R^2_m = 0.531 (95% CI: 0.529-0.533)

Ecosystem respiration: R_e ~ $T^4\sigma\ e^{\frac{-SW}{\sigma T^4}}$ + GS + ND + VPD + ALAN + (1|FLUXNET2015 site) + (1|Latitude)

Random effects	Variance	SD	Fixed effects	Estimate ±SE	T value
FLUXNET2015 site	0.278	0.527	Intercept	-4.77 ±0.115	-41.45
Latitude (°)	0.785	0.886	$T^4\sigma\ e^{-SW/\sigma T4}$	0.025 ±2.62×10^{-5}	956.61
Residual	1.43	1.20	Growing Season	0.750 ±1.82×10^{-3}	456.81
			Night Duration	-0.177 ±3.49×10^{-4}	-505.81
			VPD	-0.075 ±2.43×10^{-4}	-309.79
			ALAN	0.023 ±1.76×10^{-4}	129.97

AIC = 13,354,001; df = 8; R^2_m = 0.499; R^2_c = 0.713, n = 4,181,247; Bootstrapped R^2_m = 0.499 (95% CI: 0.497-0.501)

Supplementary Table 4. Null half-hourly ecosystem carbon flux generalised additive mixed models (GAMMs) for *NEE*, *GPP* and R_e with FLUXNET2015 site (n = 86) set as random effects. Latitude was originally allocated as a random effect but all GAMMs indicated redundancy, defined statistically as a lack of improvement in model fit and concurvity = 1, indicating complete collinearity with other smooth terms. Model diagnostics are shown for backward selection against the full model which includes explanatory variables identified in LMMs (growing season (GS), night duration (ND), vapor pressure deficit (VPD), ALAN, distance to nearest urban polygon (DtNUP)). Tensor-product interactions and stratification between terms was also tested. Model selection was based on a combination of penalised likelihood (fREML), adjusted R^2, concurvity checks and approximate significance of smooth terms. Final backward selected models were also compared with the re-introduction of additional variables, but none improved model fit. Final models were variance-weighted to control for overfitting to observations with high residual variability.

GAMMs	R^2_{adj}	fREML	Scale Est.	Concurvity	Smooth *p*-value	Parametric *p*-value
**Full model** *NEE* ~ $T^4\sigma\ e^{\frac{-SW}{\sigma T^4}}$ + GS + s(ND, by GS) + s(VPD, by GS) + s(ALAN, by GS) + DtNUP + ti(ALAN, ND, by GS)	0.56	2.92 ×10^7	19.19	Many > 0.8	All ****	DtNUP *
**Backward selected model** *NEE* ~ $T^4\sigma\ e^{\frac{-SW}{\sigma T^4}}$ + GS + s(ND) + ti(ALAN, ND)	0.538	2.94 ×10^7	20.16	All < 0.7	All ****	
**Variance-weighted model** (as above)	0.431	1.35 ×10^7	0.860	All < 0.7	All ****	Int. ns
**Final weighted model** *NEE* ~ $T^4\sigma\ e^{\frac{-SW}{\sigma T^4}}$ + s(ND) + ti(ALAN, ND)	0.430	1.35×10^7	0.877	All < 0.7	All ****	
**Full model** *GPP* ~ $T^4\sigma\ e^{\frac{-SW}{\sigma T^4}}$ + GS + s(ND, by GS) + s(VPD, by GS) + s(ALAN, by GS) + DtNUP + ti(ALAN, ND, by GS)	0.622	1.64 ×10^7	27.60	Many > 0.8	All ****	Int. ns
**Backward selected model** *GPP* ~ $T^4\sigma\ e^{\frac{-SW}{\sigma T^4}}$ + GS + s(ND) + ti(ALAN, ND)	0.603	1.66 ×10^7	28.94	All < 0.65	All ****	
**Variance-weighted model** (as above)	0.517	7.32 ×10^6	0.907	All < 0.65	All ****	
**Full model** R_e ~ $T^4\sigma\ e^{\frac{-SW}{\sigma T^4}}$ + GS + s(ND, by GS) + s(VPD, by GS) + s(ALAN, by GS) + DtNUP + ti(ALAN, ND, by GS)	0.696	6.47×10^6	1.29	All > 0.80	All ****	Int. * DtNUP *
**Backward selected model** R_e ~ $T^4\sigma\ e^{\frac{-SW}{\sigma T^4}}$ + GS + s(ND) + ti(ALAN, ND)	0.676	6.60×10^6	1.38	All < 0.75	All ****	
**Variance-weighted model** (as above)	0.613	5.75×10^6	0.914	All < 0.75	All ****	

Supplementary Table 5. Final GAMMs with selected terms as presented in Supplementary Table 4. All models were for half-hourly carbon flux measurements (μmol CO_2 m^{-2} s^{-1}), with all *NEE*, daytime *GPP* and nighttime R_e measurements (see Methods). Models include smooth terms for the Arrhenius function ($T^4\sigma\ e^{\frac{-SW}{\sigma T^4}}$, J m^{-2} s^{-1}), growing season (GS = Y: growing season, GS = N: non-growing season), ALAN, night duration (ND, hours), and tensor-product interactions between ALAN and ND. Random smooths were included for FLUXNET2015 site (n=86) and latitude (n=80), but s(Latitude) was identified as redundant, defined statistically as a lack of improvement in model fit and concurvity = 1, indicating complete collinearity with other smooth terms. Reported edf are the estimated degrees of freedom for each smooth. All smooth terms were significant (p < 0.0001: ****).

Net ecosystem exchange: *NEE* ~ $T^4\sigma\ e^{\frac{-SW}{\sigma T^4}}$ + s(ND) + ti(ALAN,ND)

Term	Estimate	T value	Smooth terms	Edf	F
Intercept	-1.11 ±0.106	-10.47****	s($T^4\sigma\ e^{\frac{-SW}{\sigma T^4}}$)	8.99	810,946****
			s(ND)	8.98	2,663****
			(ALAN×ND)	15.95	2,774 ****
			s(FLUXNET2015 site)	84.99	10,886****

R^2_{adj} = 0.430; Deviance explained = 44.9%; fREML = 1.35 ×10^7 ; n = 10,069,905

Gross primary production: *GPP* ~ $T^4\sigma\ e^{\frac{-SW}{\sigma T^4}}$ + GS + s(ND) + ti(ALAN,ND)

Term	Estimate	T value	Smooth terms	Edf	F
Intercept	1.68 ±0.206	8.14****	s($T^4\sigma\ e^{\frac{-SW}{\sigma T^4}}$)	9.00	543,337****
GS	5.11 ±0.004	1163.75****	s(ND)	8.98	21,454****
			(ALAN×ND)	15.98	1,435****
			s(FLUXNET2015 site)	84.99	10,588****

R^2_{adj} = 0.517; Deviance explained = 56.7%; fREML =7.32 ×10^6; n = 5,340,294

Ecosystem respiration: R_e~ $T^4\sigma\ e^{\frac{-SW}{\sigma T^4}}$ + GS + s(ND) + ti(ALAN,ND)

Term	Estimate	T value	Smooth terms	Edf	F
Intercept	1.98 ±0.092	21.50****	s($T^4\sigma\ e^{\frac{-SW}{\sigma T^4}}$)	9.00	118,113****
GS	0.648 ±0.002	443.60****	s(ND)	8.96	21,945****
			(ALAN×ND)	15.93	2,981****
			s(FLUXNET2015 site)	84.99	26,277****

R^2_{adj} = 0.613; Deviance explained = 63.8%; fREML = 5.75×10^6; n = 4,181,247

Supplementary Table 6. Null linear mixed effect models (LMMs) fitted to mean daily *NEE*, *GPP* and R_e observations (n = 197,247), with FLUXNET2015 site (n = 86) and latitude (n = 80) set as random effects, showing model goodness of fits with additional selected terms (growing season, continent, climate, IGBP land use classification, night duration, hour of the day, vapor pressure deficit (VPD), precipitation, ALAN, distance to nearest urban polygon (DtNUP) and proportion urban land cover in 3 and 10 km buffers of each site tested). Terms that were not selected based on model fit and parsimony conditions ($\Delta AIC_{df} < -5$ and $\Delta R^2_{mdf} > 0.01$) are not shown. Final selected model results are presented in Supplementary Table 7.

**Net ecosystem exchange (NEE)**	**df**	R^2_m	R^2_c	ΔAIC_{df}	ΔR^2_{mdf}
$GPP \sim T^4\sigma\ e^{\frac{-SW}{\sigma T^4}}$ (null)	5	0.151	0.303	0	0
+ Growing Season	6	0.275	0.418	-39,657	0.124
+ Night Duration	7	0.303	0.439	-10,773	0.028
**Gross primary production (GPP)**	**df**	R^2_m	R^2_c	ΔAIC_{df}	ΔR^2_{mdf}
$GPP \sim T^4\sigma\ e^{\frac{-SW}{\sigma T^4}}$ (null)	5	0.125	0.303	0	0
+ Growing Season	6	0.430	0.553	-100,863	+0.306
+ Night Duration	7	0.453	0.576	-12,603	+0.022
+ DtNUP	8	0.480	0.573	-13.03	+0.028
**Ecosystem respiration (R_e)**					
$R_e \sim T^4\sigma\ e^{\frac{-SW}{\sigma T^4}}$ (null)	5	0.013	0.265	0	0
+ Growing Season	6	0.362	0.553	-108,203	+0.349
+ Night Duration	7	0.427	0.624	-34,984	+0.065
+ ALAN	8	0.442	0.637	-578.56	+0.016

Supplementary Table 7. Final LMMs with selected terms as presented in Supplementary Table 6. All models were for mean daily carbon flux measurements (μmol CO_2 m^{-2} s^{-1}), with FLUXNET2015 site (n = 86) and latitude (n = 80) set as random effects in all models. Model fits are shown for each final model, including bootstrapped 95% confidence intervals from 500 semiparametric bootstrap replicates.

Net ecosystem exchange: *NEE* ~ $T^4\sigma\ e^{\frac{-SW}{\sigma T^4}}$ + Growing Season + Night Duration + (1|FLUXNET2015 site) + (1|Latitude)

**Random effects**	**Variance**	**SD**	**Fixed effects**	**Estimate ±SE**	**T value**
FLUXNET2015 site	0.610	0.781	Intercept	-5.14 ±0.115	-44.68
Latitude (°)	0.380	0.616	$T^4\sigma\ e^{-SW/\sigma T4}$	0.009 ±8.07×10^{-5}	115.97
Residual	4.10	2.02	Growing Season	-1.20±0.013	-94.06
			Night Duration	0.281 ±2.66×10^{-3}	105.28

AIC = 838,489; df = 7; R^2_m = 0.303; R^2_c = 0.439, n = 197,247, Bootstrapped R^2_m = 0.303 (95% CI: 0.300 -0.306)

Gross primary production: *GPP* ~ $T^4\sigma\ e^{\frac{-SW}{\sigma T^4}}$ + Growing Season + Night Duration + DtNUP + (1|FLUXNET2015 site) + (1|Latitude)

**Random effects**	**Variance**	**SD**	**Fixed effects**	**Estimate ±SE**	**T value**
FLUXNET2015 site	1.23	1.11	Intercept	15.52 ±0.331	46.91
Latitude (°)	3.11	1.76	$T^4\sigma\ e^{-SW/\sigma T4}$	-0.017 ±1.77×10^{-4}	-93.23
Residual	19.81	4.45	Growing Season	5.87 ±0.028	208.70
			Night Duration	-0.669 ±5.86×10^{-3}	-114.14
			DtNUP	-0.024 ±4.65×10^{-3}	-5.25

AIC = 1,149,267; df = 8; R^2_m = 0.480; R^2_c = 0.573, n = 197,247, Bootstrapped R^2_m = 0.481 (95% CI: 0.477-0.484)

Ecosystem respiration: R_e ~ $T^4\sigma\ e^{\frac{-SW}{\sigma T^4}}$ + Growing Season + Night Duration + ALAN + (1|FLUXNET2015 site) + (1|Latitude)

**Random effects**	**Variance**	**SD**	**Fixed effects**	**Estimate ±SE**	**T value**
FLUXNET2015 site	0.273	0.522	Intercept	4.98 ±0.115	43.17
Latitude (°)	0.741	0.861	$T^4\sigma\ e^{-SW/\sigma T4}$	0.004 ±5.48×10^{-5}	80.28
Residual	1.89	1.376	Growing Season	1.62 ±8.69×10^{-3}	186.44
			Night Duration	-0.355 ±1.81×10^{-3}	-195.72
			ALAN	0.022 ±9.12×10^{-4}	24.37

AIC = 686,261; df = 8; R^2_m = 0.443; R^2_c = 0.637, n = 197,247, Bootstrapped R^2_m = 0.443 (95% CI: 0.439-0.446)

Supplementary Table 8. Backward selection of GAMMs fitted to mean daily *NEE, GPP,* and R_e observations with FLUXNET2015 site (n = 86) set as random effects. Latitude was originally allocated as a random effect but all GAMMs indicated redundancy, defined statistically as a lack of improvement in model fit and concurvity = 1, indicating complete collinearity with other smooth terms. Model diagnostics are shown for backward selection against the full model which includes explanatory variables identified in LMMs (growing season (GS), night duration (ND), ALAN, distance to nearest urban polygon (DtNUP)). Tensor-product interactions and stratification between terms were also tested. Model selection for was based on a combination of penalised likelihood (fREML), adjusted R^2, concurvity checks and approximate significance of smooth terms. Final backward selected models were also compared with the re-introduction of additional variables, but none improved model fit. Final models were variance-weighted to control for overfitting to observations with high residual variability.

GAMMs	R^2_{adj}	fREML	Scale Est.	Concurvity	Smooth *p*-value	Parametric *p*-value
**Full model** *NEE* ~ $T^4\sigma\ e^{\frac{-SW}{\sigma T^4}}$ + GS + DtNUP + s(ND, by GS) + s(ALAN, by GS) + ti(ALAN, ND, by GS)	0.465	4.12 ×10^5	3.79	All > 0.8	All ****	DtNUP *
**Backward selected model** *NEE* ~ $T^4\sigma\ e^{\frac{-SW}{\sigma T^4}}$ + GS + ti(ALAN, ND)	0.423	4.12 ×10^5	4.08	All < 0.75	All ****	Intercept ***
**Variance-weighted model** (as above)	0.370	2.78×10^5	0.978	All < 0.75	All ****	Intercept **
**Full model** *GPP* ~ $T^4\sigma\ e^{\frac{-SW}{\sigma T^4}}$ + GS + DtNUP + s(ND, by GS) + s(ALAN, by GS) + ti(ALAN, ND, by GS)	0.587	5.68 ×10^5	18.56	Many > 0.8	All ****	All ****
**Backward selected model** *GPP* ~ $T^4\sigma\ e^{\frac{-SW}{\sigma T^4}}$ + GS + ti(ALAN, ND)	0.559	5.75 ×10^5	19.81	All < 0.55	All ****	All ****
**Variance-weighted model** (as above)	0.539	2.73 ×10^5	0.933	All < 0.55	All ****	All ****
**Full model** R_e ~ $T^4\sigma\ e^{\frac{-SW}{\sigma T^4}}$ + GS + DtNUP + s(ND, by GS) + s(ALAN, by GS) + ti(ALAN, ND, by GS)	0.644	3.36 ×10^5	1.76	Many > 0.8	All ****	DtNUP **
**Backward selected model** R_e ~ $T^4\sigma\ e^{\frac{-SW}{\sigma T^4}}$ + GS + ti(ALAN, ND)	0.581	3.52 ×10^5	2.07	All < 0.30	All ****	All ****
**Variance-weighted model** (as above)	0.545	2.74 ×10^5	0.936	All < 0.30	All ****	All ****

Supplementary Table 9. Final variance-weighted GAMMs with selected terms as presented in Supplementary Table 8, fitted to mean daily carbon flux measurements (μmol CO_2 m^{-2} s^{-1}) for daytime *GPP* and nighttime R_e (see Methods). Models include smooth terms for the Arrhenius function, growing season (GS = Y: growing season, GS = N: non-growing season), ALAN, night duration (ND, hours), and tensor-product interactions between ALAN and ND. Random smooths were included for FLUXNET2015 site (n=86) and latitude (n=80), but s(Latitude) was identified as redundant, defined statistically as a lack of improvement in model fit and concurvity = 1, indicating complete collinearity with other smooth terms. Reported Edf are the estimated degrees of freedom for each smooth. All smooth terms were significant ($p < 0.0001$: ****).

Net ecosystem exchange: *NEE* ~ $T^4\sigma\ e^{\frac{-SW}{\sigma T^4}}$ + GS + ti(ALAN,ND)

**Parametric effect**	**Estimate**	**T value**	**Smooth terms**	**Edf**	**F**
Intercept	0.308 ±0.099	3.11 **	s($T^4\sigma\ e^{\frac{-SW}{\sigma T^4}}$)	8.78	3174 ****
GS	-1.48 ±0.009	-152.51****	ti(ALAN×ND)	15.73	523.10 ****
			s(FLUXNET2015 site)	84.66	431.40 ****

R^2_{adj} = 0.370; Deviance explained = 38.3%; fREML = 2.78 ×10^5; n = 197,247

Gross primary production: *GPP* ~ $T^4\sigma\ e^{\frac{-SW}{\sigma T^4}}$ + GS + ti(ALAN,ND)

**Parametric effect**	**Estimate**	**T value**	**Smooth terms**	**Edf**	**F**
Intercept	2.56 ±0.120	12.33 ****	s($T^4\sigma\ e^{\frac{-SW}{\sigma T^4}}$)	8.72	2343 ****
GS	6.26 ±0.021	302.34 ****	ti(ALAN×ND)	15.79	554.70 ****
			s(FLUXNET2015 site)	84.68	399.60 ****

R^2_{adj} = 0.539; Deviance explained = 56.7%; fREML = 2.73 ×10^5; n = 197,247

Ecosystem respiration: R_e ~ $T^4\sigma\ e^{\frac{-SW}{\sigma T^4}}$ + GS + ti(ALAN,ND)

**Parametric effect**	**Estimate**	**T value**	**Smooth terms**	**Edf**	**F**
Intercept	1.80 ±0.091	19.85 ****	s($T^4\sigma\ e^{\frac{-SW}{\sigma T^4}}$)	8.24	572.60 ****
GS	1.96 ±0.007	293.28 ****	ti(ALAN×ND)	15.64	985.90 ****
			s(FLUXNET2015 site)	84.79	662.10 ****

R^2_{adj} = 0.545; Deviance explained = 56.8%; fREML = 2.74 ×10^5; n = 197,247

Supplementary Table 10. Summary of path coefficients from the final structural equation model (SEM) evaluating the relationship between annual *NEE*, *GPP* and R_e observations. The table presents unstandardized and standardized path coefficients (β), standard errors (SE), and *p*-values for each directional path in the model, alongside residual correlations. The model showed good fit (C = 29.99, df = 20, p = 0.070; AIC = 24,843; R^2_m = 0.33 and R^2_c = 0.84 for R_e; R^2_m = 0.34 and R^2_c = 0.74 for *GPP* and R^2_m = 0.77 and R^2_c = 0.83 for *NEE*).

**Response**	**Predictor**	**Estimate**	**SE**	**Std. β**	***P***
GPP	*SW*	119.54	47.29	0.224	0.0126
GPP	GS Length	162.45	18.26	0.305	<0.0001
GPP	VPD	-229.39	43.03	-0.430	<0.0001
GPP	DtNUP	-170.96	36.86	-0.321	<0.0001
R_e	*GPP*	0.357	0.026	0.401	<0.0001
R_e	*T*	28.02	8.15	0.235	0.0007
R_e	VPD	-95.64	29.29	-0.202	0.0012
R_e	ALAN	80.15	24.09	0.169	0.0010
NEE	R_e	0.637	0.026	0.760	<0.0001
NEE	*GPP*	-0.762	0.022	-1.02	<0.0001
NEE	GS Length	33.37	9.34	0.084	0.0004
Residual correlation	*SW* ~~ VPD	—	—	-0.231	<0.0001
Residual correlation	VPD ~~ ALAN	—	—	0.782	<0.0001
Residual correlation	DtNUP ~~ ALAN	—	—	-0.246	<0.0001

Supplementary Table 11. Leave-one-out sensitivity analysis of the final SEM, assessing the effects of removing each exogenous variable on model fit (*Fisher's C*, *P*, df), model parsimony (AIC), and explanatory power (R^2_m and R^2_c) for *NEE*, *GPP* and R_e. Asterisks (*) highlight models with significantly worse fit (P < 0.05, ΔAIC_{df} > 5, or ΔR^2_m / R^2_c < 0.001), suggesting high sensitivity to the excluded variable.

**SEM**	**Fisher's *C***	***P***	**df**	**AIC**	**R^2_m / R^2_c (*NEE*)**	**R^2_m / R^2_c (*GPP*)**	**R^2_m / R^2_c (R_e)**
Full model	29.99	0.070	20	24,843	0.765 / 0.830	0.344 / 0.736	0.334 / 0.840
– *SW*	24.49	0.079	19	24,857*	0.765 / 0.830	0.255 / 0.729*	0.334 / 0.840
– *T*	26.90	0.043*	19	24,859*	0.765 / 0.827*	0.344 / 0.736	0.290 / 0.827*
– DtNUP	21.15	0.173	19	24,870*	0.765 / 0.830	0.220 / 0.725*	0.334 / 0.840
– VPD	31.39	0.026*	18	24,894*	0.765 / 0.830	0.286 / 0.736*	0.259 / 0.832*
– ALAN	28.13	0.030*	19	24,860*	0.765 / 0.830	0.344 / 0.736	0.300 / 0.826*
– GS Length	43.10	0.001*	18	24,939*	0.758 / 0.825*	0.258 / 0.725*	0.334 / 0.840
