# Supplementary material for: Widespread influence of artificial light at night on ecosystem metabolism
Source: Nat Clim Chang. 2025 Nov 12;15(12):1371–7. doi: 10.1038/s41558-025-02481-0 (PMC12672368; doi:10.1038/s41558-025-02481-0)
Supplement: Supplementary file 2 — Reporting summary [file 41558_2025_2481_MOESM2_ESM.pdf]

nature portfolio

Corresponding author(s): Alice S.A. Johnston

Last updated by author(s): 30/09/2025

Reporting Summary

Nature Portfolio wishes to improve the reproducibility of the work that we publish. This form provides structure for consistency and transparency in reporting. For further information on Nature Portfolio policies, see our Editorial Policies and the Editorial Policy Checklist.

Statistics

For all statistical analyses, confirm that the following items are present in the figure legend, table legend, main text, or Methods section.

n/a	Confirmed	
☐	☒	The exact sample size (n) for each experimental group/condition, given as a discrete number and unit of measurement
☒	☐	A statement on whether measurements were taken from distinct samples or whether the same sample was measured repeatedly
☐	☒	The statistical test(s) used AND whether they are one- or two-sided<br>*Only common tests should be described solely by name; describe more complex techniques in the Methods section.*
☐	☒	A description of all covariates tested
☐	☒	A description of any assumptions or corrections, such as tests of normality and adjustment for multiple comparisons
☐	☒	A full description of the statistical parameters including central tendency (e.g. means) or other basic estimates (e.g. regression coefficient) AND variation (e.g. standard deviation) or associated estimates of uncertainty (e.g. confidence intervals)
☐	☒	For null hypothesis testing, the test statistic (e.g. F, t, r) with confidence intervals, effect sizes, degrees of freedom and P value noted<br>*Give P values as exact values whenever suitable.*
☒	☐	For Bayesian analysis, information on the choice of priors and Markov chain Monte Carlo settings
☐	☒	For hierarchical and complex designs, identification of the appropriate level for tests and full reporting of outcomes
☒	☐	Estimates of effect sizes (e.g. Cohen's d, Pearson's r), indicating how they were calculated

Our web collection on statistics for biologists contains articles on many of the points above.

Software and code

Policy information about availability of computer code

Data collection	QGIS 3.30.3
Data analysis	R version 4.2.2

For manuscripts utilizing custom algorithms or software that are central to the research but not yet described in published literature, software must be made available to editors and reviewers. We strongly encourage code deposition in a community repository (e.g. GitHub). See the Nature Portfolio guidelines for submitting code & software for further information.

Data

Policy information about availability of data

All manuscripts must include a data availability statement. This statement should provide the following information, where applicable:

- Accession codes, unique identifiers, or web links for publicly available datasets
- A description of any restrictions on data availability
- For clinical datasets or third party data, please ensure that the statement adheres to our policy

The FLUXNET2015 data analysed in this study are available at https://fluxnet.fluxdata.org/data/fluxnet2015-dataset/ (see ref. 30) and are subject to the FLUXNET data policy (https://fluxnet.org/data/data-policy). As redistribution of raw half-hourly flux data is not permitted, we provide only derived products, including daily and annual summaries, processed variables, and model outputs, which are available under a CC-BY 4.0 license on Figshare (https://doi.org/10.6084/

m9.figshare.29958455, see ref. 57). The ALAN metrics used here are available at https://doi.org/10.3390/rs9060637 (see ref. 32). Summaries for each FLUXNET site are also provided in Supplementary Table 1.

Research involving human participants, their data, or biological material

Policy information about studies with human participants or human data. See also policy information about sex, gender (identity/presentation), and sexual orientation and race, ethnicity and racism.

Reporting on sex and gender	na
Reporting on race, ethnicity, or other socially relevant groupings	na
Population characteristics	na
Recruitment	na
Ethics oversight	na

Note that full information on the approval of the study protocol must also be provided in the manuscript.

Field-specific reporting

Please select the one below that is the best fit for your research. If you are not sure, read the appropriate sections before making your selection.

☐ Life sciences ☐ Behavioural & social sciences ☒ Ecological, evolutionary & environmental sciences

For a reference copy of the document with all sections, see nature.com/documents/nr-reporting-summary-flat.pdf

Ecological, evolutionary & environmental sciences study design

All studies must disclose on these points even when the disclosure is negative.

Study description	The study compiles half-hourly net ecosystem exchange, gross primary production and ecosystem respiration measurements from 86 FLUXNET2015 sites, which were partitioned into daytime and nighttime fluxes. Annual carbon fluxes were also compiled from the FLUXNET2015 website. Factors included in this study for each site include air temperature, incoming shortwave radiation, IGBP land use classification, site latitude and longitude and date, alongside carbon fluxes. Annual ALAN metrics were compiled for each of the 86 FLUXNET site's, using latitude and longitude and filtered to only include the relevant study years for which carbon fluxes were available. Urban metrics were calculated by quantifying the proportion of land cover classified as urban within 3 and 10 km buffers around each site, using the ESA CCI Land Cover dataset, and computing the Euclidean distance (km) from site centroid to the nearest urban polygon in the Copernicus Urban Centre Database (ref. 55 in manuscript). Daily night duration at each study site was calculated as the time between astronomical sunset and sunrise (UTC) using site-specific latitude, longitude and observation dates using the suncalc package in R. Analysis, conducted in R, combined linear mixed effect models, generalized additive mixed models and piecewise structural equation models.
Research sample	The study includes the compilation of existing FLUXNET2015 datasets (acquired from https://fluxnet.org/data/ and adhering to the CC-BY-4.0 Data Policy) and publicly available ALAN datasets (https://doi.org/10.3390/rs9060637, ref. 33 in manuscript)
Sampling strategy	Sample size for each site depended on the number of measurements available for the carbon flux and fundamental constraint variables of interest.
Data collection	Data was collected from the existing FLUXNET2015 datasets provided by https://fluxnet.org/data/ and ALAN datasets provided by https://doi.org/10.3390/rs9060637. Measurements from each site were merged in to a single dataset.
Timing and spatial scale	The FLUXNET2015 data were collected between 1991 and 2014, and span a latitudinal range from 28.46 °N to 56.48 °N.
Data exclusions	NA values (-9999 values in downloaded FLUXNET2015 data) for carbon fluxes and for air temperature (TA_F) or incoming shortwave radiation (SW_IN) were excluded during calculation of the modified Arrhenius function.
Reproducibility	All statistical analyses are fully reproducible when the data and code provided are run. However, the raw half-hourly measurements cannot be provided to adhere to the data policy.
Randomization	FLUXNET2015 sites were categorised into broad climate groups (boreal, temperate, mediterranean) which included some generalisations. For instance, alpine sites were classified as boreal climates and mediterranean climates were classified based on site latitude and mean annual temperature.
Blinding	Blinding is not relevant to this study as we do not compare control and treatment groups.

Did the study involve field work? ☐ Yes ☒ No

Reporting for specific materials, systems and methods

We require information from authors about some types of materials, experimental systems and methods used in many studies. Here, indicate whether each material, system or method listed is relevant to your study. If you are not sure if a list item applies to your research, read the appropriate section before selecting a response.

Materials & experimental systems

n/a	Involved in the study
☒	☐ Antibodies
☒	☐ Eukaryotic cell lines
☒	☐ Palaeontology and archaeology
☒	☐ Animals and other organisms
☒	☐ Clinical data
☒	☐ Dual use research of concern
☒	☐ Plants

Methods

n/a	Involved in the study
☒	☐ ChIP-seq
☒	☐ Flow cytometry
☒	☐ MRI-based neuroimaging

Plants

Seed stocks

Report on the source of all seed stocks or other plant material used. If applicable, state the seed stock centre and catalogue number. If plant specimens were collected from the field, describe the collection location, date and sampling procedures.

Novel plant genotypes

Describe the methods by which all novel plant genotypes were produced. This includes those generated by transgenic approaches, gene editing, chemical/radiation-based mutagenesis and hybridization. For transgenic lines, describe the transformation method, the number of independent lines analyzed and the generation upon which experiments were performed. For gene-edited lines, describe the editor used, the endogenous sequence targeted for editing, the targeting guide RNA sequence (if applicable) and how the editor was applied.

Authentication

Describe any authentication procedures for each seed stock used or novel genotype generated. Describe any experiments used to assess the effect of a mutation and, where applicable, how potential secondary effects (e.g. second site T-DNA insertions, mosiacism, off-target gene editing) were examined.
